# Cohesin Is Limiting for the Suppression of DNA Damage–Induced Recombination between Homologous Chromosomes

**DOI:** 10.1371/journal.pgen.1001006

**Published:** 2010-07-01

**Authors:** Shay Covo, James W. Westmoreland, Dmitry A. Gordenin, Michael A. Resnick

**Affiliations:** Laboratory of Molecular Genetics, National Institute of Environmental Health Sciences (NIEHS), National Institutes of Health (NIH), Research Triangle Park, North Carolina, United States of America; Brandeis University, United States of America

## Abstract

Double-strand break (DSB) repair through homologous recombination (HR) is an evolutionarily conserved process that is generally error-free. The risk to genome stability posed by nonallelic recombination or loss-of-heterozygosity could be reduced by confining HR to sister chromatids, thereby preventing recombination between homologous chromosomes. Here we show that the sister chromatid cohesion complex (cohesin) is a limiting factor in the control of DSB repair and genome stability and that it suppresses DNA damage–induced interactions between homologues. We developed a gene dosage system in tetraploid yeast to address limitations on various essential components in DSB repair and HR. Unlike *RAD50* and *RAD51*, which play a direct role in HR, a 4-fold reduction in the number of essential *MCD1* sister chromatid cohesion subunit genes affected survival of gamma-irradiated G_2_/M cells. The decreased survival reflected a reduction in DSB repair. Importantly, HR between homologous chromosomes was strongly increased by ionizing radiation in G_2_/M cells with a single copy of *MCD1* or *SMC3* even at radiation doses where survival was high and DSB repair was efficient. The increased recombination also extended to nonlethal doses of UV, which did not induce DSBs. The DNA damage–induced recombinants in G_2_/M cells included crossovers. Thus, the cohesin complex has a dual role in protecting chromosome integrity: it promotes DSB repair and recombination between sister chromatids, and it suppresses damage-induced recombination between homologues. The effects of limited amounts of Mcd1and Smc3 indicate that small changes in cohesin levels may increase the risk of genome instability, which may lead to genetic diseases and cancer.

## Author Summary

The cellular concentrations of individual proteins are expected to be kept within an optimal range, but protein expression is often stochastic. Some proteins are known to be in limiting amounts, so that even modest reduction can lead to malfunction. Within the network of genes that determine genome stability, proteins that are limiting impose a risk for the cell, because fluctuation in their amounts may start a cascade of genomic alternations that will influence many biochemical pathways either under normal growth conditions or in response to chromosome damage. We sought to identify genes that are limiting for DSB repair by lowering the dosage of key genes from 4 to 1 in tetraploid *Saccharomyces cerevisiae* strains. We found that the complex that holds sister chromatid cohesion together (cohesin) is limiting in DSB repair. In addition, when it is reduced modestly, recombination between homologous chromosomes is highly increased, suggesting that the risk for loss of hetrozygosity (LOH) is increased too. These results should also be considered in light of increasing evidence that copy number variation can impact cellular function.

## Introduction

Genome stability is maintained by a network of proteins that ensure faithful DNA replication and efficient response to DNA damage. Variation in levels of proteins across the cell cycle, between tissues and even through natural fluctuations are common [1], [2], [3] and could influence genome stability especially for proteins that are present in limiting amounts. Proteins with limited expression are likely to be weak links in genome maintenance and, therefore, could be risk factors in disease, especially cancer predisposition, when combined with environmental stress. This could be particularly important for the cases where small, environmentally relevant amounts of genotoxins inhibit a mutation avoidance repair system [4]. Even a cell with WT genotype may be at risk for genome instability due to fluctuation in expression of limiting proteins.

Many genes are involved in spontaneous and damage-induced homologous recombination (HR) ensuring efficiency and accuracy. The repair of double-strand breaks (DSBs) by HR is an evolutionarily conserved process (for review, see [5]) and is generally considered error free since it uses information from an undamaged DNA template. However, since HR can also occur between related as well as identical sequences it can lead to genomic instability through loss-of-heterozygosity (LOH) and nonallelic recombination between repeats across the genome, which can result in chromosome rearrangements [6], [7]. These changes are often detected in genetic disorders, cancer and during evolution (discussed in, [8], [9], [10]).

Mutations in HR components can lead to genome instability and cancer predisposition [11]. Increased genome instability can also result from changes in the amounts of wild type gene products functioning in HR. In yeast, a genome wide analysis identified 178 genes with haplo-insufficiency causing increased chromosome loss in the heterozygote state [12]. Included was *RAD55*, which is directly related to HR; it showed both chromosomal instability and sensitivity to DNA damage when heterozygous. Haplo-insufficiency for several human genes leads to DNA damage sensitivity, genome instability and/or cancer susceptibility, suggesting they are present in amounts that are limiting for HR [13], [14].

We sought to identify more proteins that are present in limiting amounts for HR-mediated DSB repair and to assess the consequences of reduced levels. The identification of proteins that when limiting affect genome stability can be accomplished through manipulation of gene dosage in polyploid cells. Small variations in the amount of a protein can be accomplished with tetraploid strains of the budding yeast *Saccharomyces cerevisiae* where gene dosage can be varied over a factor of 4 from one (simplex) to four copies (tetraplex; referred to as WT) by deleting copies of the gene from homologous chromosomes. This scheme provides the opportunity to address the relationship between gene dosage and biological consequences for many genes. It also enables studies reduced amounts of essential gene products. Importantly, unlike other systems for down-regulating proteins, the amount of a protein can be reduced without affecting the coding sequence or other transcription/translation controls of the remaining alleles. This approach was used for the yeast photolyase DNA repair gene *PHR1* [15] which can reverse UV-induced pyrimidine dimers and the *RAD52* gene [16] which is essential for recombinational repair of DSBs [17].

We applied the reduced gene dosage approach to three genes that impact HR: *RAD50*, *RAD51*, and *MCD1*. The MRX complex in yeast, which includes Rad50, is responsible for DSB recognition and DNA resection, the first step in HR and in DNA damage signaling at site-specific and in damage-induced genome wide DSBs ([18], [19] and references therein). The Rad51 protein which is directly involved in recombination including homology search and formation of joint molecule (for review see, [20]) was previously suggested to be present in limiting amounts [21]. We found that changes in levels of Rad50 and Rad51 did not affect the response to ionizing radiation.

We also investigated the consequences to genome stability of reducing the dosage of genes affecting sister chromatid cohesion. While not directly involved enzymatically in HR [22], the sister chromatid cohesion complex (cohesin) that includes Mcd1, Smc3, Smc1 and Irr1 is important in DSB repair in haploid yeast cells ([23] and for review, see [24]). Following induction of DSBs, cohesin is recruited to DSBs via the DNA damage response pathway [22], [25]. The cohesin becomes cohesive even at undamaged sites of the genome [26], [27]. Although cohesin facilitates DSB repair between sister chromatids, its impact when homologous chromosomes are present is unknown. Recombination between sister chromatids is generally acknowledged to be more efficient than between homologous chromosomes [28] suggesting that cohesin inhibits recombination between homologous chromosomes. In this sense, cohesin might suppress opportunities for LOH as well as nonallelic recombination and chromosome rearrangements involving repeated DNAs. Previously it was shown that cohesin can influence the pattern of recombination induced by a single DSB in a plasmid based assay [29]. However, since cohesin is an essential gene and viable mutants are likely to be sensitive to ionizing radiation it is not known what role it might play in maintaining recombination fidelity when survival is high.

Here we show that even a modest reduction in the level of cohesin dramatically increases the ability of γ–radiation to induce recombination between homologous chromosomes in the G_2_ but not the G_1_ phase of the cell cycle even at low radiation doses when survival is high. This finding, which also extends to UV-induced recombination, suggests that cohesin confines recombinational repair to sister chromatids even in the absence of DSBs, thereby reducing the risk of genome instability.

## Results

### Identifying limiting factors in DSB repair using a gene dosage approach

**Figure 1:**
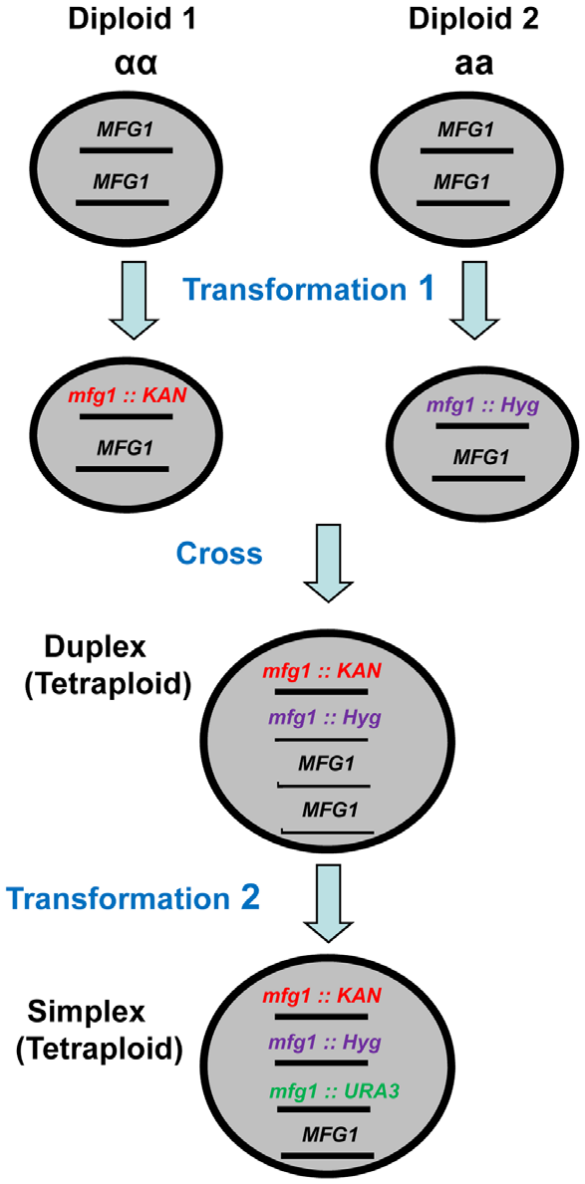
Development of tetraploid simplex strains. Within Diploids 1 and 2, which have opposite mating types, one copy of the gene of interest (*MFG 1*, *i.e.*, *my favorite gene*) was inactivated in each diploid by transformation with kanamycin or hygromicin cassettes that target deletions. The resulting diploids were crossed to create Met^+^ (see Materials and Methods) Kan^R^ and Hyg^R^ tetraploids with two copies (duplex) of *MFG1*. Finally, a third copy of *MFG1* was inactivated by transforming the duplex strain with a *URA3* targeting cassette.

In order to identify factors that are limiting for DSB repair, pairs of tetraploid strains were produced that were simplex or WT for genes of interest and examined for IR sensitivity. To develop the simplex strains, diploids of opposite mating types were created and transformed with gene inactivation cassettes containing different antibiotic resistance markers as described in Figure 1. The diploids were crossed to yield tetraploid strains with only two functional copies (duplex). Tetraploids were confirmed by i) loss of mating ability, ii) presence of resistance to G418 and hygromicin antibiotics and iii) methionine prototrophy due to complementation of *met2* and *met6* mutants (see Materials and Methodss). Finally, a simplex strain was created by inactivating one of the two remaining functional genes in the duplex strain. Genotypes were confirmed by PCR at all steps in construction.

### Decrease in Mcd1, but not Rad50 or Rad51, enhances sensitivity and reduces DSB repair

**Figure 2:**
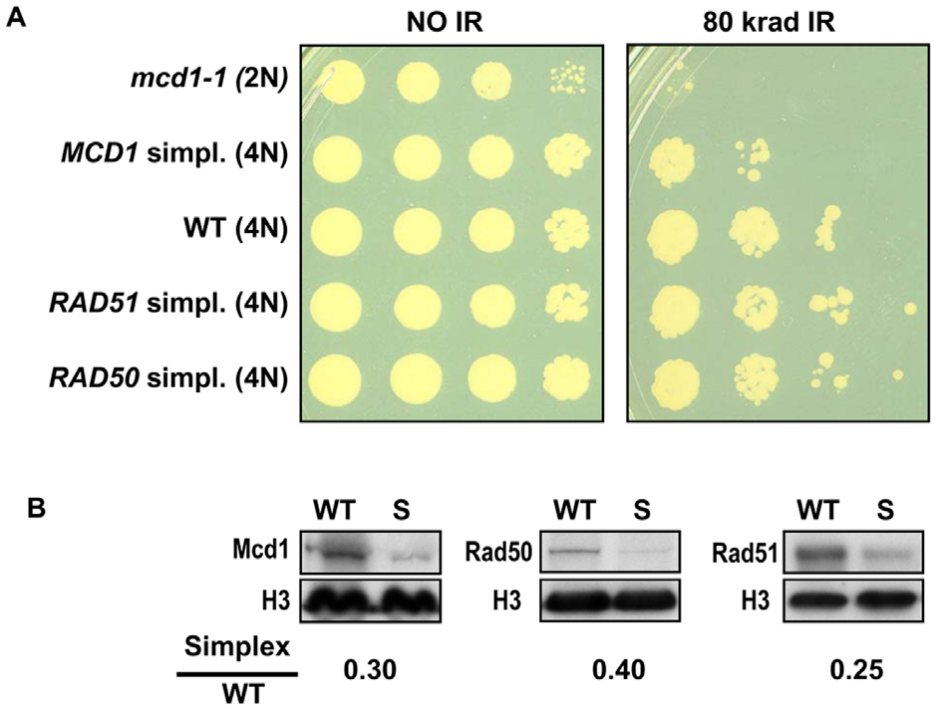
Cells that are simplex for *MCD1*, but not *RAD50* or *RAD51*, are sensitive to ionizing radiation. (A) Late logarithmically growing cells (5×10^7^ cells/ml) of tetraploid strains with 4 copies (WT) or one copy (simplex) of *MCD1*, *RAD50* and *RAD51* as well as *mcd1-1* diploid cells were spotted on YPDA plates. The control and irradiated plates (80 krad) were incubated for two days at 30°C. (B) Logarithmically growing cultures of WT, and simplex strains (presented as “S”) for *MCD1*, *RAD50* and *RAD51* were harvested in SDS PAGE sample buffer and separated on SDS PAGE. Protein amounts were determined by western blot analysis using antibodies; histone H3 (presented as “H3”) was used as a loading control. The normalized intensity of each protein in the simplex strain was divided by the normalized intensity in WT to give the simplex/WT ratio.

Changes in gene dosage of either *RAD51* or *RAD50* did not affect gamma sensitivity (Figure 2) after exposure to 80 krad. However, there was a marked increase in sensitivity for the *MCD1* simplex compared to WT strains, which was not attributable to growth effects (Figure 2 and Figure S1). In contrast, a temperature sensitive *mcd1-1* diploid cell shows high sensitivity to IR and slower growth based on the appearance of small colonies after 2 days of growth. As expected, reduction in the expression of each of the respective proteins in the logarithmically growing simplex strains was close to 4-fold (considering variability of Western blot measurements) in comparison to WT as shown in Figure 2B. Thus, it appears that unlike Rad50 and Rad51, the Mcd1 protein is limiting for cellular responses to gamma radiation.

**Figure 3:**
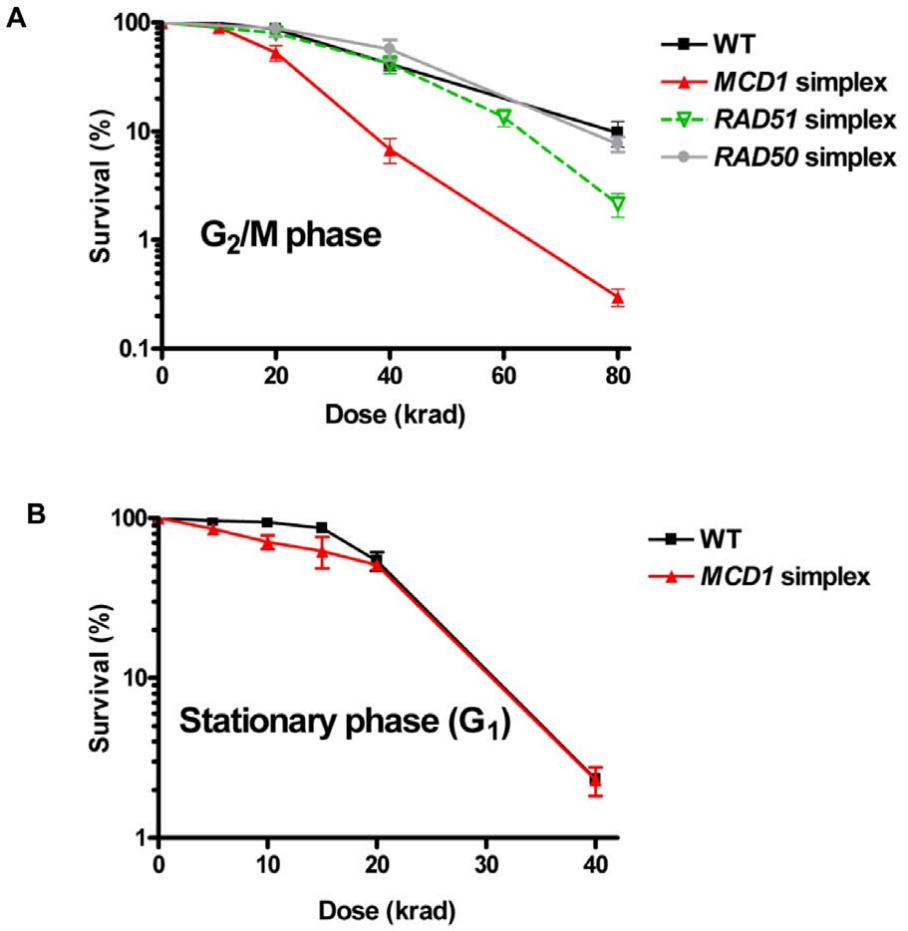
*MCD1* simplex cells are more sensitive than the WT to IR as G_2_/M but not G_1_ (stationary) phase cells. (A) Nocodazole-arrested WT *RAD50*, *RAD51* and *MCD1* simplex cells (G_2_/M) or (B) WT and *MCD1*cells from 3 day stationary cultures (G_1_) were irradiated with the indicated doses. Cells were spread on YPDA (described in Materials and Methods under “Nocodazole arrest, gamma irradiation, and post irradiation incubation”) or complete synthetic media plates (there was no difference in survival between the two types of media). Survival was determined after 2–3 days. Survival was determined from at least 6 cultures for each genotype; error bars correspond to the standard error (SEM).

To address more precisely the importance of cohesin in cells containing sister chromatids and to determine if there are subtle effects in *RAD50* and *RAD51* simplex strains, cells were gamma irradiated after nocadazole induced G_2_/M arrest. As shown in Figure 3A, the *MCD1* simplex strain was clearly more susceptible to IR than WT. There was a 2-fold increase in the dose-modifying factor (*e.g.*, the same killing was achieved with half the dose) which corresponds to a large difference in survival over the range of 20 to 80 krad (Figure 3A; 52% for the *MCD1* simplex vs 87% for the WT at 20 krad; *p* = 0.009, n = 13). While survival of the *MCD1* simplex strain was lower than WT, it could still tolerate many DSBs (over 100, based on estimates from [18]). Since the genomes of tetraploid yeast cells are somewhat unstable, we considered the possibility that a portion of the *MCD*1 simplex population had gained an extra copy of *MCD1*. To rule this out, we determined, the survival of 10 *MCD*1 duplex and 19 *MCD*1 simplex isolates after 80 krad exposure of cultures arrested by nocadazole. There was no overlap between the duplex and the simplex cells. The survivals of all the *MCD1* simplex cultures were 3–300 fold less than the median survival of the duplex *MCD1* strains (data not shown). Thus, if there are some cells in a simplex population that have an additional copy of *MCD1*, their frequency is small and would only be expected to result in an underestimation of the induced recombination frequencies (see results and discussion below).

The role of Mcd1 in resistance to IR was also confirmed with homozygous diploids carrying the *mcd1-1* temperature-sensitive allele when cells were plated at the semi-permissive temperature 32°C after irradiation (Figure S2). Neither *RAD50* nor *RAD51* simplex strain showed any change from WT strain in the dose modifying factor (Figure 3A), although *RAD51* simplex strain were somewhat more sensitive to IR at high doses. Based on these results, we chose to focus the rest of this study on cohesin.

### A reduced level of Mcd1 does not affect radiation sensitivity of G1 cells, but does affect sensitivity and DSB repair in G_2_/M cells

Since sister chromatid cohesion is established during S phase and disrupted during anaphase we asked whether cohesin affects the response to IR during the G_1_ stage of the cell cycle, when cells lack sister chromatids. Previous studies with yeast have been restricted to survival or DSB repair measurements with haploid cells, which would lack any opportunity for repair between homologous chromosomes. The absence of repair of radiation-induced DSBs by nonhomologous end-joining [18], unlike mammalian cells, render yeast a good model for addressing defects in homologous recombination. The *MCD1* simplex and WT cells were grown for 3 days to stationary phase (>90% G_1_ cells, based on cell morphology) and exposed to IR. No significant difference was observed between WT and *MCD1* simplex cells at this stage. Importantly, the response of *MCD1* simplex cells irradiated at G_2_/M or as stationary cells (primarily G_1_), was comparable to that of WT cells in stationary cells (Figure 3). These results suggest that the cohesin function associated with sister chromatids has little role in DSB repair that might occur between homologous chromosomes in G_1_ cells.

**Figure 4:**
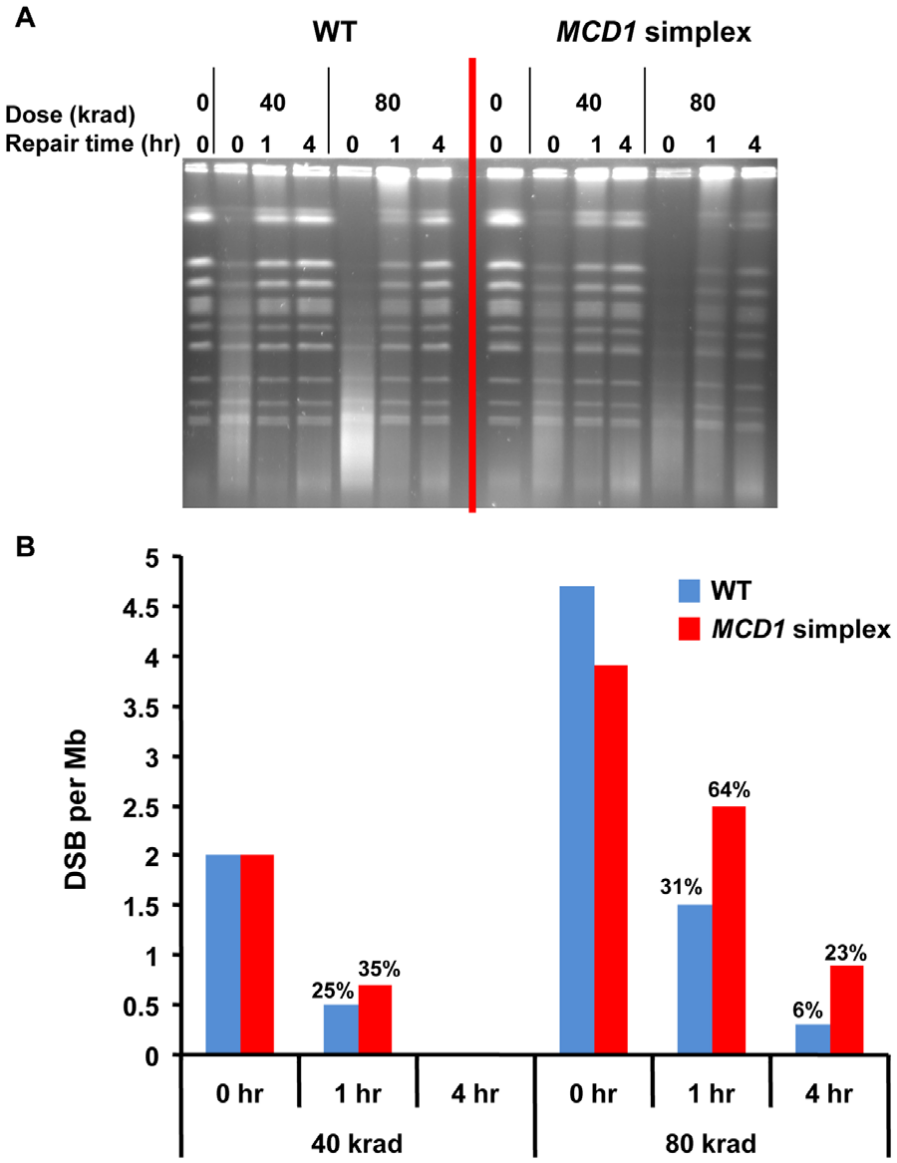
DSBs are repaired slowly in *MCD1* simplex as compared to WT cells. Logarithmically growing *MCD1* simplex and WT strains in YPDA medium at 30°C were arrested in nocodazole for 2.5 hours and irradiated with the indicated dose (See Materials and Methods). Samples were taken before and immediately after radiation. Cells were re-suspended in warm YPDA medium containing nocadazole and incubation was continued. Samples were taken at 1 and 4 hr. (A) The chromosomal DNA was displayed using PFGE. (B) The induction of DSBs and subsequent repair was calculated from the intensity of the retained chromosomes. A detailed description of the preparation of DNA plugs, PFGE conditions and DSB quantification is presented in Materials and Methods.

We examined directly the impact of decreased levels of Mcd1 on DSB repair in the G_2_/M cells using pulsed field gel electrophoresis (PFGE) [6], [18]. PFGE separates individual chromosomes on the basis of size so that gamma induced DSBs and repair can be readily assessed (Figure 4). The efficiency of DSB repair is determined by an analysis of restitution of full size chromosomes during post-irradiation incubation (see Materials and Methods). While repair was detected, the *MCD1* simplex strain clearly exhibited reduced repair capacity in comparison to WT cells as shown for cells irradiated with 80 krad, corresponding to ∼600 DSBs/cell (Figure 4), [6], [18]. The reduced levels of Mcd1 significantly affected the rate of repair at 1 to 4 hr post-irradiation incubation (see Figure 4). For example, within 1 hr after IR the WT cells repaired ∼70% of the DSBs induced by 80 krad while half as many were repaired in the *MCD1* simplex strain (Figure 4B). Increasing post-irradiation incubation time to 4 hr led to more repair in the WT and *MCD1* simplex cells; however, there were still about 4 times more unrepaired breaks in the *MCD1* simplex than the WT cells (23% vs 6%). At a lower dose (40 krad; Figure 4), reduced levels of Mcd1 had less of an impact consistent with the smaller differences in killing (Figure 3A). We note the limited ability of the PFGE repair assay to detect small differences in DSBR capacity. This is relevant to considerations of IR induced lethality since unrepaired DSBs appear to have a dominant effect on cell killing [30].

Thus, we establish that the level of Mcd1 is critical both for efficient repair of DSBs and maintaining resistance to radiation in G_2_/M cells.

### Reduction in cohesin subunits Mcd1 and Smc3 increases IR–induced recombination between homologous chromosomes in G2 cells

**Figure 5:**
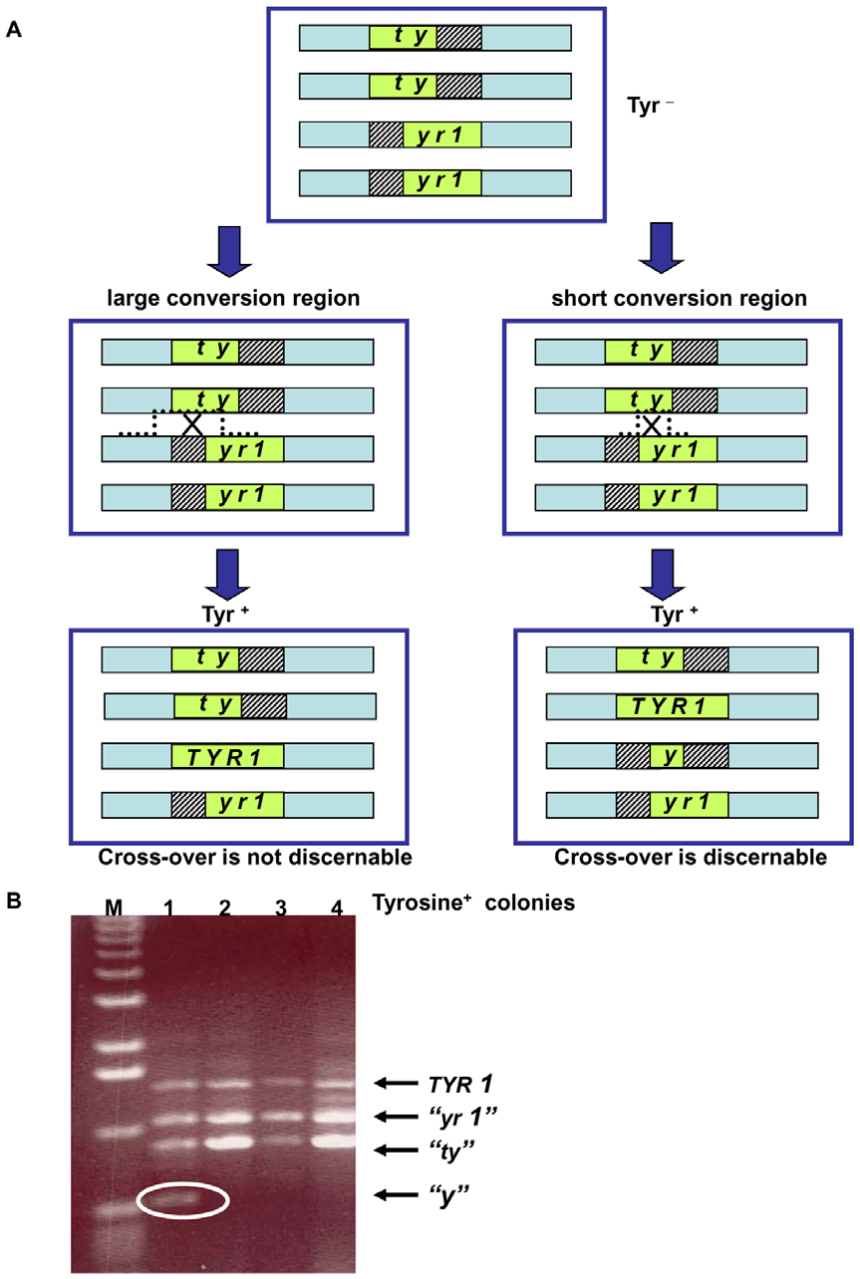
Genetic reporter for recombination between heteroalles residing on homologous chromosomes in tetraploid strains. (A) The tetraploid cells carry two versions of chromosome II with deletions within the *TYR1* ORF: the “*ty*”-allele contains a 5′ portion of the *TYR1* ORF (nucleotides 1–700) and the “*yr1*”-allele contains a 3′ portion (nucleotides 300–1358). The green box represents the ORF of the *TYR1* gene; the striped boxes represent the missing DNA sequences of the mutants; and the light blue rectangle represents the area of homology between the chromosomes. Note: for simplicity only one of each pair of sister chromatids of the G_2_/M cells are presented (thereby, having the appearance of G_1_ cells). (For a complete description of recombination and segregation at mitosis see Figure S7.) Gene conversion with or without crossing-over as well as reciprocal exchange between heteroalleles can generate Tyr^+^ cells. Recombination leading to Tyr^+^ can generate different combinations of *TYR1* alleles within the resulting tetraploid cell. A large conversion tract will result in 3 types of alleles regardless of associated crossing-over: the original truncated parental heteroalleles and the *TYR1^+^* converted allele. A reciprocal exchange that occurs at short conversion region will yield a forth allele “y” retains only a small portion of the gene. Depending on segregation of sister chromatids at mitosis, half the Tyr^+^ recombinants that arise by a reciprocal exchange would not possess the “y” allele (for details see Figure S7). (B) The 4 alleles described in (A) can be distinguished by size of PCR products using the following primers: 5′GAATACCGTAGCACTTGAAGGAAAGAGGACAGCATATCCA 5′CACAAAAGAAGGCCTAATATTATAGGAAATCAGCATTAAAAAC. The allele sizes are 1360 bp (*TYR1*), 1060 bp (“*yr1*”), 700 bp (“*ty*”) and 400 bp (“*y*”). Presented are PCR products of the *TYR1* locus from 4 colonies obtained after UV irradiation (40 J/m^2^) of the *MCD1* simplex strains. Tyr^+^ colonies that result from a reciprocal exchange event can be identified by the presence of a “*y*” allele (encircled). The “M” corresponds to DNA molecular size markers.

The decreased DSB repair in *MCD1* simplex cells arrested at G_2_/M suggests that there might be a change in interactions between homologous chromosomes. To address directly recombination between homologous chromosomes, we developed the genetic reporter described in Figure 5. The tetraploid cells carry two versions of chromosome II where two of the four chromosomes contain the 5′ portion of the *TYR1* and the other two carry the 3′ portion. The 3′ and 5′ truncations have a 400 bp overlap; such that homologous recombination can lead to Tyr^+^ cells (see Figure 5 and Materials and Methods). The *TYR1* recombinants are likely to arise through a gene conversion process that covers only one of the alleles either associated or not associated with cross-over (Figure 5A). They could also occur by a DSB in the homologous region between the two heteroalleles generating a tract that ends between the alleles, which could result in a reciprocal exchange (Figure 5). We assume that changes in the frequency of Tyr^+^ recombinants are directly correlated with changes in number of interactions between homologous chromosomes.

**Figure 6:**
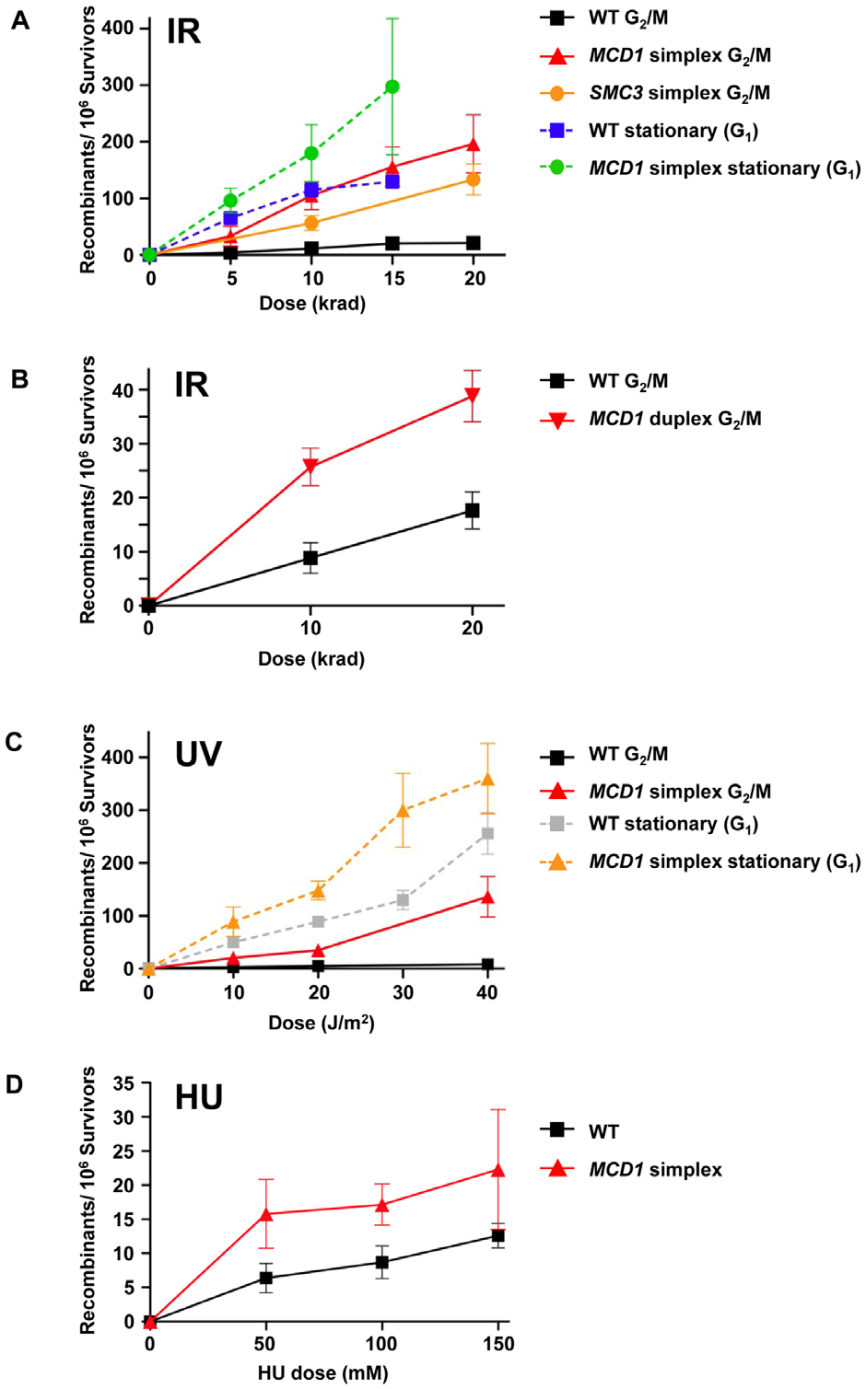
Cohesin is limiting in the suppression of damage-induced recombination. (A) Induction of recombination by ionizing radiation of nocodozole-arrested WT, *MCD1* and *SMC3* simplex cells and stationary WT and *MCD1* simplex cells. After irradiation, cells were plated to synthetic complete (SC) or SC lacking tyrosine (SC-Tyr) and incubated for 2–3 days. Shown are the net recombination frequencies (induced minus “no irradiation”). (B) IR-induced recombination in nocodazole arrested *MCD1* duplex cells (as a comparison, the WT data was pooled from panel A and four more WT cultures that were done side-by-side). (C) UV-induction of recombination in nocodozole-arrested and stationary WT and *MCD1* simplex cells. (D) Induction of recombination by hydroxyurea. Logarithmically growing WT or *MCD1* simplex cells were treated with HU overnight. Cells were then spread on complete and Tyr^−^ plates. Presented are induced recombination frequencies (the frequency measured after HU treatment minus the frequency measured without treatment; see legend to Figure S5 for a detailed description). Recombination frequencies were obtained from at least 8 cultures for each genotype and for each DNA damaging agent.

We found that the spontaneous rates of Tyr^+^ recombination were not affected by the level of Mcd1. The median rates for the simplex and the WT *MCD1* strains were 2.5×10^−6^ (1.1–2.9×10^−6^; 95% confidence interval) and 1.5×10^−6^ (1–2×10^−6^; 95% confidence interval). Exposure to IR increased the frequencies of Tyr^+^ recombinants in G_2_/M arrested cells in all strains examined. The efficiency of induction in *MCD1* simplex cells (∼5–10×10^−6^ recombinants/survivor/krad) was approximately 10-fold greater than in WT cells over a range extending from sublethal doses to ∼50% survival at 20 krad (Figure 6A; see Figure 3A for survival), even though DSBR is efficient (Figure 4). (Based on an induction efficiency of 0.07 DSB/mb/krad [18] there are sufficient DSBs to account for the observed recombinants even if all events are generated by DSBs in the 400 nt overlap region.) Since the recombination assay scores infrequent events (<0.1% of the population), it is possible that some of the Tyr^+^ colonies were not *MCD1* simplex. In order to estimate a change in *mcd1* deletion alleles, 160 presumptive *MCD1* simplex Tyr^+^ colonies arising after nocodazole arrest and 20 krad treatment, were replica-plated to the appropriate media to verify the presence of the simplex markers (G418 and Hygromycin resistance and Ura^+^ phenotype). Only 4 colonies lost one of these markers (2.5%), suggesting that most of the colonies were actually *MCD1* simplex.

The *MCD1* duplex cells (two functional gene copies out of 4, see Figure 1) also showed a significant elevation in IR-induced recombination frequency (Figure 6B). For example, for WT and *MCD1* duplex cells irradiated with 10 krad the induced frequencies were 10±3×10^−6^ and 28±3×10^−6^, respectively (*p* = 0.013, n = 9). Responses were very different with G1 stationary cells where HR interactions are restricted to homologous chromosomes. The induction of recombination in the stationary cells was marginally influenced by *MCD1* gene dosage (<2 fold; Figure 6A). Interestingly, the recombination frequencies in the WT cells irradiated at stationary stage matched the HR response of the simplex strain irradiated at G_2_/M (Figure 6A).

The cohesin complex itself appears to be limiting since simplex strains of *SMC3*, another member of the complex, also showed elevated IR-induced HR between homologues in G_2_/M cells (Figure 6A). At 10 and 20 krad, corresponding to 80% and 70% survival, respectively, the Tyr^+^ recombinant frequency in the *SMC3* simplex strain was about 5-fold higher than the WT.

The impact that a reduced level of Mcd1 has on recombination between homologues was not specific to tetraploid cells. We also addressed the consequences of lowering Mcd1 function in a diploid strain. As shown in Figure S3, the levels of IR-induced recombination in G_2_/M arrested WT tetraploid is not higher than in diploid cells, suggesting that the additional chromosomes alone do not increase opportunities for recombination. Since the temperature sensitive *mcd1-1* mutation has frequently been employed to address the role of cohesin in haploid cells [31], we also investigated IR-induced recombination in a homozygous *mcd1-1* diploid strain at the semi-permissive temperature of 32°C. The level of induced *TYR1* recombination was 3-fold higher (18±3×10^−6^ vs 60±10×10^−6^; *p*-value 0.002, n = 6) than in the WT diploid following exposure to 20 krad (there was a 5-fold difference in survival; see Figure S2). However, the impact on recombination was less than for the *MCD1* simplex strain (discussed below). Since the *MCD1* duplex showed higher recombination frequencies than the WT tetraploid (Figure 6B), it was expected that an *MCD1* hetrozygous diploid would have an elevated frequency of induced recombination between homologous chromosomes. Indeed, the induced frequency for the *MCD1* hetrozygote was slightly higher than for the WT diploid following exposure to 20 krad: 28±3×10^−6^ and 18±3×10^−6^, respectively. A similar difference was observed at 40 krad (43 vs 28 recombinants/10^−6^ survivors, respectively). For both doses the differences were statistically significant based on a one-tailed *t* test (*p* = 0.0194 and 0.0273 for 20 and 40 krad, respectively; n = 10). The differences between homozygous and heterozygous *MCD1* diploids are smaller than the differences between WT tetraploid and *MCD1* duplex, suggesting that there is an additional component(s) that further sensitizes tetraploid cells to cohesion defects. In support of this view, *mcd1-1* tetraploid cells are inviable even at a temperature that enabled growth of the corresponding diploid [32].

In summary, the *MCD1* simplex strain shows lower global DSB repair capacity but higher radiation-induced recombination frequencies between homologous chromosomes than the WT cells. We conclude that the limiting levels of cohesin are sufficient to direct repair of gamma induced DSBs towards sister chromatids and that reductions in cohesin open opportunities for recombination between homologous chromosomes.

### Decreased *MCD1* gene dosage also increases UV– and HU–induced recombination

UV radiation can induce recombination between sister chromatids and homologous chromosomes [33]. It does not generate DSBs directly, although they might arise through repair of closely spaced lesions on complementary strands or during replication. Surprisingly, the reduction in *MCD1* gene dosage resulted in UV-induced increases in HR frequencies in G_2_/M cells comparable to those for IR (Figure 6A and Figure 6C): 20±3/10^6^ survivors for the simplex strain vs 2±0.7/10^6^ survivors for WT at 10 J/m^2^ (p = 0.0001). The difference was increased to 20-fold, reaching 132±38 recombinants/10^6^ survivors at 40 J/m^2^ vs 8±2 recombinants/10^6^ for WT cells (Figure 6C).

Based on experiments with *rad52* haploid cells that are unable to repair DSBs, the differences between the *MCD1* simplex and WT is not attributable to UV being able to generate DSBs directly or indirectly in the G_2_/M cells. While survival after 40 J/m^2^ UV irradiation of *rad52* cells was 15%, survival after 20 krad was less than 0.1% indicating that many more DSBs occur when cells are irradiated with 20 krad than 40 J/m^2^ UV. The recombination is likely to arise in the G_2_/M cells rather than in the subsequent S phase. UV- induced recombination in WT stationary cells was over 10-fold greater than in G_2_/M (Figure 6C) suggesting that UV lesions generated at G1 or entering the next S phase are still highly recombinogenic even in WT cells. Surprisingly, the recombination frequency for UV-irradiated stationary phase *MCD1* simplex cells was only 2-fold higher than for WT cells (Figure 6C), far less of an effect than for cells irradiated at G_2_/M.

The low UV-induced recombination rates for WT cells irradiated at G_2_/M could stem from very efficient nucleotide excision repair that removes UV lesions at G_2_, possibly suggesting that the high recombination rates of *MCD1* simplex might be due to reduced efficiency in removal UV lesions. However, the *MCD1* simplex strain was not sensitive to UV. Survival following exposure to 10 and 20 J/m^2^ at G_2_/M was 100% for both the WT and *MCD1* simplex strains; even at 40 J/m^2^ the survival was similar for the *MCD1* simplex and WT strains (68±20% and 89±13%, respectively). Also, irradiation of unsynchronized cells showed no difference between WT and *MCD1* simplex cells even at high UV doses (Figure S4).

The DNA synthesis inhibitor hydroxyurea (HU) can generate stalled replication forks leading to DSBs that can be rescued by recombination [34]. We asked whether differences in *MCD1* levels could influence HU-induced recombination between homologous chromosomes. Logarithmically growing cells were treated with HU overnight and recombination between homologous chromosomes was determined. Growth inhibition ranged between 25% and 90% (50 to 150 mM) and was somewhat higher in the simplex as compared to the WT strain (Figure S5). At these doses there was induction of recombinants in both the *MCD1* simplex and WT strains (Figure 6D); however, the frequencies (<20 recombinants/10^6^ survivors) were much lower than for IR- and UV-induced recombination (Figure 6). Reduction in the level of Mcd1 resulted in only a small (∼2-fold) increase in HU-induced recombination over the WT strain.

### Reduced Mcd1 levels open the genome to gene conversion and crossing over between homologous chromosomes

Since gene conversion may be associated with crossing-over at the chromosome level, increases in Tyr^+^ prototrophs - are likely to reflect increases in cross-overs and, therefore, LOH. If reciprocal crossing-over occurs in the G_2_ stage of the cell cycle, half the events would result in chromosomes with long stretches of LOH, depending on segregation of the sister chromatids while crossing-over in G_1_ would not yield LOH (see Figure S6).

Reciprocal exchange (RE) products containing the “*y*” allele result from cross-overs that fall between the two *tyr1* heteroalleles (Figure 5A). These can be identified by PCR genotyping (Figure 5B) as short fragments distinct from the wild type recombinant allele (*TYR1*) and the alleles that were unaffected by the recombination event (“*tyr*” and “*yr1*”). If a crossing-over event leading to Tyr^+^ occurs in G1 or in S phase cells prior to replication of the *TYR1* region, the “y” allele would appear in all progeny cells. However, for cross-overs in G_2_/M only half the “y” alleles would be recovered because of sister chromatid segregation (see Figure S7); therefore, the observed frequency of “*y*” alleles among the Tyr^+^ recombinants is a minimal estimate of the actual RE frequency. We note that the above PCR based assay only detects cross-overs with a short conversion tract, while events with a long tract will not be discernable (Figure 5) (see also [35] for conversion tract length in mitotic crossing over).

**Figure 7:**
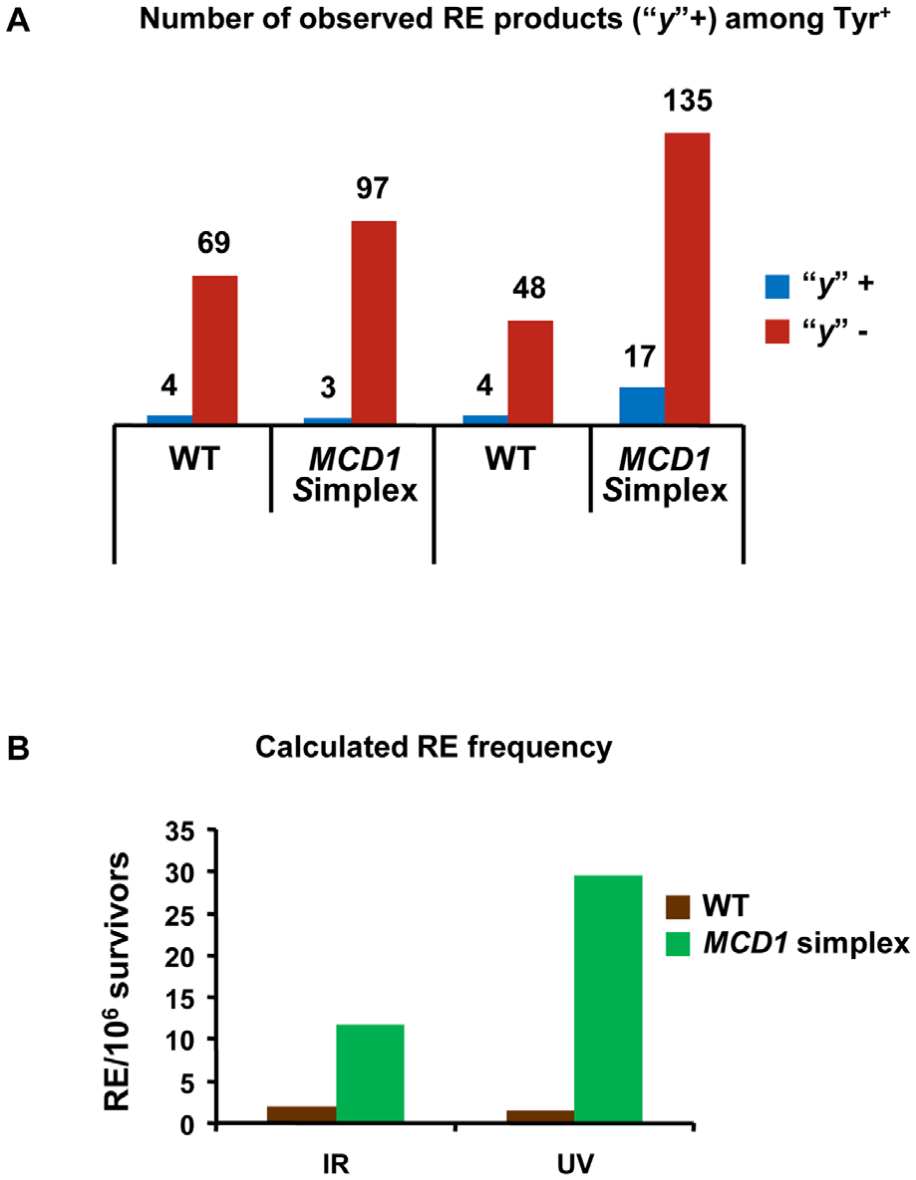
Both reciprocal and non-reciprocal exchanges are found in DNA damage–induced *TYR1* recombinants. (A) Minimal estimation of reciprocal exchanges (RE) was obtained by PCR amplification of *TYR1* locus of Tyr^+^ colonies (details in text and in Figure 5) (The numbers of events were “*y*” was identified or not are presented). (B) Calculation of excepted frequency of RE in *TYR1* locus of irradiated cells. The minimal estimate for RE frequency among Tyr^+^ recombinants (7A) was multiplied by 2, assuming that all events occurred in G_2_ (Figure S7) and then was multiplied by the frequency of Tyr^+^ induced recombinants as measured in Figure 6.

We determined the cross-overs among the Tyr^+^ recombinants after exposure of G_2_/M cells to 20 krad IR or 40 J/m^2^. The “*y*” allele was observed in a small fraction of the Tyr^+^ recombinants appearing after IR and UV exposure of the WT and simplex strains (Figure 7A). The minimum frequency of REs among Tyr^+^ recombinants did not differ significantly between the *MCD1* simplex and WT Tyr^+^ strains. Based on the overall recombination frequencies presented in Figure 6A and Figure 6C and assuming recombination occurred in the G_2_/M cells, the expected induced RE frequency in *MCD1*simplex is much higher than in WT cells, as described in Figure 7B. Thus, while a reduction in the amount of Mcd1 does not change the recombination fate in terms of cross-overs *vs* no cross-overs, we suggest that the a reduced level of Mcd1 places the genome at considerable risk for both IR and UV induced gene conversion and crossing-over.

## Discussion

### The gene dosage approach to identifying limiting factors in genome stability

**Figure 8:**
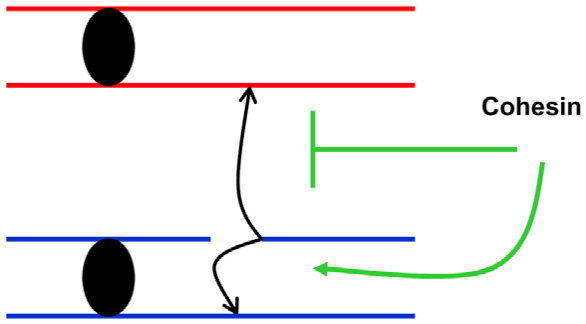
Cohesin channels HR to sister chromatids. Presented is a model in which cohesin-mediated interactions between sister chromatids reduces opportunities for damage-induced recombination between homologous chromosomes.

In order to address the consequences of moderate changes in key proteins responsible for DSB repair we developed tetraploid strains with changes in dosage of the corresponding genes. Using survival response to DNA damage as a screening tool, Mcd1 was identified as a limiting factor in DSBR unlike Rad50 and Rad51 whose complete elimination confers extreme sensitivity to IR. This approach by itself may provide tools for identifying targets that could be used for radiotherapy sensitization (see below). More importantly, this approach allowed us to focus on cohesin as a limiting factor in maintaining genome stability. We note that other proteins are also limiting for genome stability maintenance in yeast and mammalian cells as demonstrated for several genes that exhibit haplo-insufficiency [12], [13]. We were able to show that recombination between homologous chromosome is highly increased in a cohesin simplex strain (Figure 6) suggesting that cohesin channels DSB repair to sister chromatids and suppresses recombination between homologous chromosomes (Figure 8).

As illustrated in Figure S6 restricting recombinational repair to sister chromatids reduces the likelihood of LOH as well as nonallelic recombination, thereby decreasing opportunities for damage-induced variations in genomic structure [6]. The combination of LOH and nonallelic recombination can be a powerful source of carcinogenesis. While it is well established that cohesin facilitates DSB recombinational repair through stabilization of sister chromatid interactions, suggestions that there is a corresponding decrease in opportunities for DSB repair through homologous recombination have lacked experimental support, especially since experiments were done in haploid yeast. Furthermore, there has been no discussion of a potential impact on recombination induced by other agents, particularly those that do not generate DSBs. We have demonstrated a dramatic (nearly 10-fold) IR-induced increase in recombination between homologues even at low, sublethal doses (5–10 krad, Figure 6A and Figure 3A) under conditions of moderately reduced levels of cohesin and normal mechanisms of cellular expression. Previous experiments have utilized temperature sensitive cohesin mutants of the essential *MCD1* gene, which grow poorly and are radiation sensitive at semi-permissive temperatures (Figure 2, Figure S2). The recombination frequencies of the *mcd1-1* strain are also greater than for WT. However, the recombination frequency for *mcd1-1* at 20 krad (20% survival) is comparable (60/10^6^ survivors) to that estimated for the simplex irradiated with one third the dose (Figure 6A), corresponding to 100% survival (Figure 3A). Also, the *MCD1* simplex strain had a growth rate comparable to WT (Figure S1). Taken together, we suggest that the amount of cohesin is limiting for suppression of recombination between homologous chromosomes. We speculate that there is enough cohesin to hold the sister chromatids after DNA replication, but the non-cohesive reservoir of cohesin in the simplex strain is limiting such that it cannot suppress DNA damage-induced events that occur at G_2_ [27].

The biological importance of a 3-fold reduction in the amount of a protein (Figure 2B) leading to a 10-fold increase in recombination frequency may lie in the stochastic pattern of protein expression where 2–3 fold changes in the amount of proteins appear to be relatively common [2]. For most proteins which are not limiting, such changes are not expected to affect the biological outcome but here we show that a small perturbation in cohesin may place a cell at risk for genome instability.

The reduction in gene dosage and protein levels in *RAD50* or *RAD51* simplexes strains did not lead to IR sensitivity. The lack of difference in sensitivity for *RAD51* may be related to the much larger number of molecules per cell: 7000 molecules of Rad51 vs 1000 Mcd1 in logarithmically growing haploid cells [1]. However, expectations based strictly on number of molecules present must be balanced against number of molecules needed for function. For example, the Rad51 repair unit is a multiprotein, single-stranded DNA filament that is likely restricted to regions of DNA undergoing repair. Similarly, even though the number of Rad50 molecules is comparable to that for Mcd1 (∼800 per cell, [1])restricting these molecules to sites of damage may limit the need for Rad50. Interestingly, deletion of the *RAD50* gene results in hyper-recombination between homologous chromosomes [36] which can be explained by reduced recruitment of cohesin to DSBs [22]. The *RAD50* simplex did not exhibit hyper-recombination frequencies (data not shown), suggesting there is enough Rad50 also for cohesin recruitment. The cellular requirements for cohesin are likely much larger given that these molecules are utilized in sister chromatids across the genome. The mean distance between cohesin binding sites is 11 kb [37], [38] corresponding to around 1000 binding sites in the genome. Also, large amounts of cohesin are recruited directly to DSBs following DNA damage [22] and “noncohesive” cohesin complexes become “cohesive” at sites distal to DSBs [26], [27]. Limiting amounts of cohesin raises the question of why not more. Possibly, too much cohesin may increase the risk of nondisjunction at mitosis, a view that is supported by the antiestablishment activity of the Rad61-Pds5-Scc3 complex towards cohesin in G_2_ [39], [40].

While there have been suggestions that tetraploid and diploid yeast may differ in ability to maintain their genomes [32], the survival responses to IR are comparable on a per lesion basis from diploids to tetraploid cells (summarized in [41]). Furthermore, we found that IR-induced recombination between homologous chromosomes of G_2_/M arrested diploid and tetraploid cells did not differ significantly (Figure S3).

### A general role for Mcd1 in confining recombination to sister chromatids

We found that Mcd1 suppresses UV- as well as IR-induced recombination between homologous chromosomes. Surprisingly, the UV-induced frequencies in G_2_/M cells were increased nearly 20-fold in simplex *MCD1* cells as compared to WT cells (Figure 6C). For UV, the increased recombination is unlikely to be related to DSBs. First, the recombination frequency is similar for 40 J/m^2^ UV and 20 krad IR. At this dose of ionizing radiation, DSBs are readily detected, while UV-induced DSBs would be rare. Second, Lettier *et al.* [42] observed that most UV-induced recombination events are independent of DSB repair since they occur in a *rad52* mutant that is completely lacking in DSB repair. Until now, the link between cohesin and homologous recombination was strictly based on the relationship between DSB induction and recruitment of cohesin to the break site. From our results we conclude that cohesin restricts potential recombinational interactions induced by ionizing and UV recombination to sister chromatids (Figure 6). We consider it likely that recombination induced by other agents would be similarly affected. Cohesin may accomplish sister chromatid preference simply by holding chromatids in close vicinity at normal cohesion attachment sites [37], [38] such that the undamaged sister becomes the preferred recombination partner. In addition, cohesin may channel recombination to sister chromatids because it is recruited directly to DSBs. Exposure of single strand DNA at a DSB was shown to be important for recruiting cohesin; however, single strand DNA intermediates are found in other DNA repair pathways including repair of UV lesions. In addition, rare DSBs associated with UV damage might lead to greater amounts of cohesion between sister chromatids across the genome as demonstrated for a site-specific single DSB [26], [27].

The effects of HU and the role of *MCD1* gene dosage on recombination differed considerably from IR and UV. HU-induced recombination was only marginally elevated in the *MCD1* simplex compared to WT (Figure 6D) strain. While HU can cause fork collapse, it might be counteracted by a back-up mechanism(s) that would also be anti-recombinogenic, for example, by Srs2 helicase recruitment via PCNA sumoylation [43].

### Implications for limited cohesin complex and restriction of recombination to sister chromatids

The cohesin complex and its functions are evolutionarily conserved across eukaryotes (for review see [24], [44] and references within). Mammalian cohesin is recruited to DSBs and is part of the ATM signal transduction and important for survival after IR. [45], [46]. In addition, the Smc3 cohesin subunit is acetylated to establish cohesin, both in yeast and human cells [47], [48]. Therefore, cohesin function in DSBR is probably conserved. It will be interesting to determine if cohesin is limiting for responses to DSB inducing agents in mammalian cells. If this is the case, then cohesin might be a useful target during cancer treatment for sensitizing cells to radiation and other drugs that break DNA. Unlike fully differentiated cells, cancer cells spend more time in G_2_ and S phase, when recombination is highly efficient. Targeting cohesin might be especially efficient when combined with cell cycle inhibitors that cause G_2_ arrest. It is interesting that mutations in cohesin and related genes were found in many cancer cells that show chromosome instability (CIN). In addition, reduction of the amount of cohesin using RNAi leads to a CIN phenotype in cells with a near diploid genome. Included among the CIN events were the development of tetraploid genomes [49]; hence, a primary defect in cohesin may generate tetraploid cells with further defects in cohesion and genome stability.

While the present results indicate a general role of cohesin in control of HR, our overall approach can provide useful insights into genome dynamics as well as genetic processes associated with tetraploidy. Tetraploid cells are common among eukaryotes and during evolution [50] and show unique characteristics regarding chromosome dynamics. In yeast, polyploid cells exhibit increased genome instability in comparison to diploids [32] which makes them an interesting model for a complex genome. Importantly, mammalian hepatocytes, frequently give rise to polyploids [51]. It is worth noting that hepatocytes are continuously exposed to genotoxic insults and polyploidy is often associated with the carcinogenesis process [52]. Therefore, the damage-inducible increase in recombination observed in *MCD1* simplex cells might result in further genome instability in natural or transformed tetraploid cells.

Finally, we describe here an experimental design that can be used to search for subtle changes in essential and nonessential factors that are limiting for genome stability. Reduction in these factors can synergize with modest (*i.e.*, high survival) levels of genotoxic stress to dramatically increase genetic change. Importantly, our approach utilizes normal, wild type proteins and native gene expression regulation thereby eliminating the uncertainty associated with mutations and variations in gene expression. Tetraploids provide a wider opportunity to vary gene dosage as compared to the simple homozygote-heterozygote approach in diploids and may be more suited for addressing implications of copy number variation, as found in the human genome [53].

## Materials and Methods

### Yeast strains

For a list of strains, see Table 1 below. Each simplex strain described in the table represents the genotype of at least 2 more independent isolates that originated from 2 independent duplex parents. Haploid strains were derivatives of E134 [54] and its *met2* and *met6* derivatives DAG 647 and DAG 645 respectively. *ura 3–52* has been replaced by a complete deletion that gave rise to strains CS1004 and CS1006 (see below). CS1004 (relevant genotype *MATα met 6*-DEL) was transformed with CORE cassette [55] targeted to the 3′ end of *TYR1*, starting at nucleotide 700 of the ORF. Briefly, the following primers were used to amplify G418^R^ in tandem with the *URA3* cassette from pCORE [55] in order to create a 5′ *tyr1* allele.

**Table 1:** Intermediate steps in strain construction and final strains.

Strain name	Description	Genotype
CS 1004	Starting haploid type 1 *met6-DEL*	*MATa ade5-1 his7-2 leu2-3,112 trp1-289 ura3-Del met6-DEL*
CS 1006	Starting haploid type 2 *met2-DEL*	*MATa ade5-1 his7-2 leu2-3,112 trp1-289 ura3-Del met2-DEL*
CS 1061	3′ *tyr1* allele	As CS 1006 with tyr1 300–1359 *trp1*-*DEL*
CS 1064	5′ *tyr1* allele	As CS 1004 with tyr1 1–700 *trp1*-*DEL*
CS 1120	*mcd1-1*	As CS 1061 but *mcd1-1*
CS 1122	*mcd1-1*	As CS 1064 but *mcd1-1*
CS 2050	(Diploid)	As CS 1061 but *MATaα*
CS 2052	(Diploid)	As CS 1064 but *MATaα*
CS 2064	Starting diploid 1 (Figure 1)	As CS 2050 but *MATαα*
CS 2065	Starting diploid 2 (Figure 1)	As CS 2052 but *MATaa*
CS 2200	*MCD1* hetrozygote (Diploid)	As CS2064 but MCD1/mcd1::KAN
CS 2222	*MCD1* hetrozygote (Diploid)	As CS2065 but *MCD1/mcd1*::Hygromycin
CS 2274	*SMC3* heterozygote (Diploid)	As CS2064 but *SMC3/smc3*::KAN
CS 2277	*SMC3* heterozygote (Diploid)	As CS2065 but *SMC3/smc3*::Hygromycin
CS 2107	*RAD50* heterozygote (Diploid)	As CS2065 but *RAD50/rad50*::KAN
CS 2208	*RAD50* heterozygote (Diploid)	As CS2064 but *RAD50/rad50*::Hygromycin
CS 2251	*Rad51* heterozygote (Diploid)	As CS2064 but *RAD51/rad50*::Hygromycin
CS 2255	*rad51* homozygote (Diploid)	As CS2065 but *rad51*::KAN*/rad51*::*URA3*
CS 2054	WT (Diploid)	Cross CS1061XCS1064
CS 2259	*mcd1-1* (Diploid)	Cross CS1120XCS1122
CS 4021	WT tetraplex (Tetraploid)	Cross of CS2064XCS2065
CS 4175	*MCD1* duplex (Tetraploid)	*MCD1*/*MCD1*/*mcd1*:: Hygromycin/*mcd1*::KAN (Cross of CS2222XCS2200)
CS 4229	*SMC3* duplex (Tetraploid)	*SMC3*/*SMC3*/*smc3*:: Hygromycin/*smc3*::KAN (Cross of CS2274XCS2276)
CS 4143	*RAD50* duplex (Tetraploid)	*RAD50*/*RAD50*/*rad50*:: Hygromycin/*rad50*::KAN Cross of (CS2107XCS2208)
CS 4207	*MCD1* simplex (Tetraploid)	*MCD1*/*mcd1::URA3*/*mcd1*::Hygromycin/*mcd1*::KAN (based on CS 4175)
CS 4238	*SMC3* simplex (Tetraploid)	*SMC3*/*smc3:URA3*/*smc3*:: Hygromycin/*smc3*::KAN (based on CS 4229)
CS 4157	*RAD50* simplex (Tetraploid)	*RAD50*/*rad50*::*URA3*/*rad50*:: Hygromycin/*rad50*::KAN (based on CS 4143)
CS 4240	*RAD51* simplex (Tetraploid)	Cross CS2251XCS2255

5′ATCTATTCGAACAAGTGGCATGTTTACGCAGGATTAGCCATAACAAACCCAAGTGCACAT-GAGCTCGTTTTCGACACTGG and 5′TTATGTATTTCTTTTTTCAGCGGCCGAACGGTCACTAGAATGACTCAGAATGGTTTTTAT-TCCTTACCATTAAGTTGATC. In parallel the CS1006 (relevant genotype *MATa met 2*-DEL) was transformed with a CORE cassette targeted to the 5′ part of the *TYR1* (nucleotide #1 at ORF) in order to create a 3′ *tyr1* allele. This was done by amplifying CORE cassette with primers 5′ATGGTATCAGAGGATAAGATTGAGCAATGGAAAGCCACAAAAGTCATTGGTATAATTGGT-GAGCTCGTTTTCGACACTGG and 5′TGTGTATCAGCGGAATCTTGTCATGCTCTTCATATGTCAAATAAACTTGCTTGCTTACTTTTC-TCCTTACCATTAAGTTGATC.

After selection of the CORE integrants, the CORE was removed [55] from CS1004 (*met6*-DEL) using oligonucleotides 5′TGGCATGTTTACGCAGGATTAGCCATAACAAACCCAAGTGCACATATAAAAACCATTCTGAGTCATTCTAGTGACCGTTCGGCCGCTGAAA and 5′TTTCAGCGGCCGAACGGTCACTAGAATGACTCAGAATGGTTTTTATATGTGCACTTGGGTTTGTTATGGCTAATCCTGCGTAAACATGCCA.

The CORE was removed from CS1006 derivative (*met2*-DEL) by introducing a portion of the *TYR1* gene PCR fragment defined by primers 5′TGAAGGAAAGAGGACAGCATATCCACTTGATAAACAAAGTATTTACCCAAGGACTGCGACATCATTACCGTGCATTCCCTTCATG and 5′GCCACTTGTTCGAATAGATTCTTAGTGATATATTAACTTTCACATTTTCT.

Loss of CORE was identified by conversion of strain to G418 sensitive and 5FOA resistant. At the end of this stage, two sets of strains were created; CS1004 derivative with *met6* DEL and with *tyr1* allele of 1–700 bp and CS1006 derivative with *tyr1* allele 300–1359 bp.

Diploid cells were made from derivatives of the above (Strains CS 1061 and CS 1064 see Table 1) by introducing plasmid (YEp-HO) encoding HO endonuclease under its native promoter to the haploid strains. Non-mating cells were identified as potential diploids and confirmed by ability to sporulate. Each a/α diploid was then transformed with a pGal-HOT plasmid were HO is inducible by galactose [56]. Cells were grown 6 hr on galactose containing media to induce mating type switching. *MAT*aa and *αα* cells were identified by mating test. The aa and αα strains were isolated from each set of haploid strains (*met* 6-DEL 5′ *tyr1* allele and *met 2*-DEL 3′ *tyr1* allele). The *MAT*aa and *αa* cells served as Diploid 1and 2 as shown in Figure 1.

WT tetraploid cells were obtained by crossing several diploid isolates with opposite mating strains and complementing *met* mutations. Cells were selected on media lacking methionine and confirmed to be non-mating. They also exhibited spontaneous and UV induced Tyr+ recombination.

### *MCD1* simplex strain formation

The following oligonucleotides were used to create G418^R^ and Hygromicin^R^ cassettes that could be targeted to *MCD1* open reading frame using plasmids pFA6 and pAG32 respectively: 5′TCCATAACAAAAAAGGACTGGTCAAAGAAAAGACAACTCAATTGCACAATTACTTTACAAGAAACACGACA-CGTACGCTGCAGGTCGACGGATCCCC and 5′TTAAAGTCTTTGATCTATATATGCATCAGCTTATTGGGTCCACCAAGAAATCCCCTCGGCGTAACTAGGTT-ATCGATGAATTCGAGCTCGTTTTCGG.

The *MCD1* heterozygote diploid was created by transforming Diploid 1 and 2 cells with the targeted G418^R^ and Hygromicin^R^ cassettes, respectively. Independent *MCD1* heterozygote isolates derived from Diploids 1 and 2 were crossed to create *MCD1* duplexes (two WT alleles with one G418^R^ and one Hygromicin^R^ replacement alleles; see Figure 1). Duplexes were transformed with a *URA3* cassette that was targeted to an internal (23 aa in frame) portion of the open reading frame by amplifying *URA3* gene from pRS306 using primers 5′TGGTTACAGAAAATCCTCAACGTCTTACTGTTTTAAGACTTGCCACCAATAAGGGTCCATTAGCACAAATACAGAG-CAGATTGTACTGAGAGTGCACC and 5′TAAGCATTGATAAACCTTTCAAATAGTGCAGGTTTGGCGTCTATTTTAATATTTCCGAATGCTTCTGTTTG-CGCATCTGTGCGGTATTTCACACCGC.

Ura^+^ transformants were confirmed to be *MCD1* simplex if they maintained G418^R^, Hygromicin^R^, were non-mating and Tyr^+^ recombinants could be induced. In addition genomic DNA was purified from the putative simplex and *MCD1* locus was PCR using the flanking primers 5′GTCGAGAAAATCGCGTCTTTC and 5′AGAAAATTTCGGCTTCACCG.

Since the *MCD1* ORF size is almost identical to the G418^R^ cassette, another set of PCR derived constructs was developed using a primer within the cassette (5′CGTACGCTGCAGGTCGAC) and a primer outside the ORF (5′AGAAAATTTCGGCTTCACCG). This analysis revealed two PCR products corresponding to G418^R^ and Hygromicin^R^ cassettes. Immediately after PCR verification of the simplex genotype, patches were stored at −70°C. At the beginning of each experiment, cells were streaked from the frozen stock for single colonies which were tested for the presence of the simplex markers.

### *SMC3* simplex strain formation

*SMC3* simplex strains were created similarly using primers: 5′CATCTTTAAACAGTTTCACCATTTTTTTACAAGACGACCTGCTGGAGTAACGGTAATAGTTCACGTCTGCA-CGTACGCTGCAGGTCGACGGATCCCC and 5′CAGTACCTCTGGGAACTAATCTTTCAAAAACAGCTTCAAAGTTTTCAGAAACCTTTTGGAAAGTAGAATCA–ATCGATGAATTCGAGCTCGTTTTCGA to create G418 and Hygromycin resistant cassettes directed to the ORF as well as primers 5′ATGTATATCAAAAGGGTGATAATTAAGGGTTTTAAAACCTACAGGAACGAAACCATTATTGATAATTTCT-CAGAGCAGATTGTACTGAGAGTGCACC and 5′ACCGCGTTAACCTTTTGTTGTTTTAACTTAACAATTAGATCTTGAATTGAGTCTTTAGATTCATCCAGCTC-CGCATCTGTGCGGTATTTCACACCGC to amplify pRS306 to create a *URA3* cassette targeted to the ORF. The simplex was verified by primers flanking the locus: 5′ CATCGAAGTGTACACCTGTCACAT and 5′ GAAAAGTAATCTTTTTTGTACGTCG.

### *RAD50* simplex strain formation

*RAD50* simplexes were made in a manner similarly to above. Oligonucleotides 5′TTTCACGGCTTTGCCTTGT and 5′TCAAAGGTGCTTACGTGCTTG were used to amplify the flanking region around the *RAD50* locus in a null strain from the *Saccharomyces cerevisiae* deletion library (G418 cassette replaced ORF). Both Diploids 1 and 2 were transfected with a G418 cassette and after obtaining heterozygote diploid isolated, the G418 cassette of one of the diploids was switched to Hygromicin resistance. The two heterozygote diploids were crossed and the tetraploid duplex was transformed with a *URA3* cassette targeted to an internal portion of the ORF by amplifying the *URA3* gene from pRS306 using the following primers 5′TCTATTCAGGGCATACGGTCTTTTGACTCCAATGATAGGGAAACTATTGAATTTGGCAAGCCTCTGACTTC-AGAGCAGATTGTACTGAGAGTGCACC and 5′TATCGACCCACTCAATTTGTGATTTTTGCCTATCATCTCTCTTGACTTTGAAGAAGTGATCAGTAAATGCCG-CGCATCTGTGCGGTATTTCACACCGC. Simplex strains were selected as described above.

### *RAD51* simplex strain formation

Construction of the *RAD51* simplex was done by sequential transfection of Diploid1 with G418 and *URA3* cassettes, thereby replacing two copies of the gene. Diploid 2 was transfected with a Hygromicin cassette targeted into the ORF of the gene. G418 and Hygromicin cassettes were made based on strains from a *Saccharomyces cerevisiae* deletion library using primers 5′ TTGAGCATTCCCTGAGCATT and 5′TCCCCTAAAAGGATAAAGCCG. *URA3* cassette was created by amplifying pRS306 using primers 5′CATATATCAGAGTCACAGCTTCAGTACGGGAACGGTTCGTTGATGTCCACTGTACCAGCAGACCTTTCACCAGAGCAGATTGTACTGAGAGTGCACC and 5′GGTCACCAACACCATCTTCATAGATCGCGAACACACATTCAGCCTCTGGTAAGCAAGGTGAGTCAACAAC-CGCATCTGTGCGGTATTTCACACCGC. The *RAD51* simplex strain was created by crossing the transformed Diploids 1 and 2.

### *mcd1-1* diploid strain formation

CS1061 and CS1064 (a and α haploids) that contain the same types of *tyr1* truncation alleles described above crossed to yield WT diploid. Same haploid strains were transformed with an Age1 cut pVG257 [31] to yield the *mcd1-1* strain. The *mcd1-1* cells were verified by sequencing and later crossed to the opposite mating counterpart to create a diploid *mcd1-1* strain.

### Western blot analysis

The WT, *MCD1* simplex, *RAD50* simplex or *RAD51* simplex cells were grown overnight and diluted to fresh media and grown to for 3 hr in 30°C and then harvested. Before Cells were washed with double-distilled water and 2×10^7^ cells were re-suspended in 0.3ml SDS-running buffer, boiled for 10 min and centrifuge 5 minutes 13,000 rpm. Typically 40 µl supernatant was loaded per lane (corresponding to ∼2.5×10^6^ cells). Following electrophoresis, the gel was transferred to a membrane using a semi-dry transfer apparatus for 105 min., according to manufacturer's instructions using a PVDF membrane (Invitrogen Carlsbad, CA). All antibodies were diluted 1∶2000 except anti Rad51 that was diluted 1∶5000. Anti Mcd1 antibody was kindly provided by Dr. Alexander Strunnikov [31]. Anti yRad50 antibody sc32862 and anti yRad51 antibody sc33626 were from Santa Cruz Biotechnology (Santa Cruz, CA). The anti yHistone 3 antibody was ab1791 from Abcam (Cambridge, MA).

### Nocodazole arrest, gamma irradiation, and post irradiation incubation

The details of nocodazole arrest, and gamma irradiation have been described [6], [18]. Briefly, nocodazole (20 µg/ml, final concentration) was added to cells that were growing logarithmically at 30°C in YPDA media (1% yeast extract, 2% Bacto-Peptone, 2% dextrose, 60 µg/ml adenine sulfate). G_2_ arrest was monitored by cell morphology. Cells were collected by centrifugation, washed and re-suspended in ice-cold sterile water. The cell suspensions were kept on ice while being irradiated in a ^137^Cs irradiator (J. L. Shepherd Model 431) at a dose rate of 2.3 krads per minute. Irradiated cells were harvested by centrifugation and resuspended in YPDA at 30°C with nocodazole for post-irradiation incubation.

### Pulsed field electrophoresis (PFGE) procedures

PFGE procedures were done as previously described [18]. Briefly, Contour-clamped Homogeneous Electric Field (CHEF) systems were used for electrophoresis of yeast chromosomes in this study. Using a CHEF Mapper XA system (Bio-Rad, Hercules, CA). These plugs were prepared in 0.5% LE agarose (Seakem, Rockland, ME) using 1–2×10^7^ G2-arrested cells per 100 ul plug. They were cut to a thickness of ∼2 mm and loaded in the bottom of a preparative well so that the entire DNA migrated very close to the bottom surface of the CHEF gel. PFGE running conditions were according to the CHEF auto-algorithem separates DNA's in the 250–1600 kb range.

### Stained-gel, multiple band method for quantitation of DSBs

To quantify DSBs in irradiated samples, pulsed-field gels were stained with SybrGold (Invitrogen, San Diego, CA) and photographed using a GelLogic200 imaging system (Eastman Kodak, Rochester, NY). Bands were measured using Kodak MI software (version 4.0) and the data were exported into Microsoft Excel (version 11.5.3) for further manipulations to determine DSBs. More details on the analysis are found in Figure S1A of [18]. Briefly, for each band corresponding to a complete unbroken Chromosome Y (any chromosome), the fraction of chromosomes remaining unbroken (*F_ChrY_*) after a given dose is simply the net intensity of the band divided by the net intensity of the corresponding band in the 0 krad control lane. From the Poisson distribution, the average number of DSBs (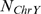) is given by the formula:
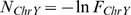



Plotting the experimentally determined values of *N* (number of breaks per chromosome) vs Molecular Weight for each chromosome band from a given dose results in an approximate straight line whose slope is in units of DSBs/mb and is independent of the total amount of DNA loaded in each lane as long as enough DNA is loaded for accurate detection of the bands. For details see reference [18]. The experimentally determined values of the slope for a given dose are highly reproducible.

### PCR identification of cross-over and non cross-over *TYR1* recombinants

Tyr^+^ cells were grown over-night in 30°C in YPDA in deep well 96 plates with shaking. Genomic DNA was purified using DNeasy of Quiagen (Valencia, CA) and amplified using primers 5′ GAATACCGTAGCACTTGAAGGAAAGAGGACAGCATATCCA and 5′ CACAAAAGAAGGCCTAATATTATAGGAAATCAGCATTAAAAAC.

## Supporting Information

Figure S1 *MCD1* simplex and WT (tetraploid) strains have comparable growth rates. Overnight cultures of WT and *MCD1* simplex cells (2–4×10^7^ cells/ml) were diluted 100-fold into fresh YPDA medium Samples were collected, diluted and plated to YPDA after 12 and 24 hr. The culture density was calculated at each time point and the relative increase (compared to time “0”) was determined, results are combined from 4 different cultures of each genetic background.
(0.20 MB TIF)

Figure S2 *mcd1-1* diploids are sensitive to IR at semi-permissive temperature.
The temperature sensitive *mcd1-1* diploid cells were grown at permissive temperature (23°C) and arrested at G_2_/M with nocodazole for 3 hr. Survival was determined for cells that were irradiated, plated to YPDA plates and incubated at semi-permissive temp (32°C). The plating efficiency without irradiation of *mcd1-1* at 32°C was 35% of that at 23°C. The same procedure was used with a WT diploid strain where no differences in plating efficiencies between 32° and 23°C were observed. Results are combined from 6 cultures of each genetic background.
(0.17 MB TIF)

Figure S3 Recombination between homologous chromosome is similar for WT diploid and tetraploid cells arrested in G_2_/M. Cells were arrested with nocodazole as described in Figure 6 and Materials and Methods and irradiated with the indicated doses. The data for the tetraploid was taken from Figure 6B (at least 12 cultures were analyzed). Six diploid cultures were analyzed.
(0.16 MB TIF)

Figure S4 *MCD1* simplex and WT strains exhibit similar UV sensitivity in asynchronous irradiated culture. Six late logarithmically growing cultures (2–4×10^7^ cells/ml) of WT and *MCD1* simplex cells were diluted 1∶20,000 and pronged using a pronging that delivers1 µl per drop and 121 drops per plates on YPDA plates (described online at http://m.pu.ru/images/stories/Perfect%20order%20plating.html). Cells were irradiated at the indicated doses and colonies were counted after 3 days.
(0.22 MB TIF)

Figure S5 Hydroxurea induced growth inhibition of WT and *MCD1* simplex strains. Stationary cultures were diluted to fresh YPDA medium and grown for 3 hr. The culture was divided into 4 equal parts and HU was added to a final concentration of 0, 50, 100 or 150 mM. Cells were grown overnight in the presence of HU then collected, diluted and spread on to synthetic complete media. Relative growth inhibition was determined from the number of colonies arising after the various treatments. This was the same procedure used to determine *TYR1* recombination, described in Figure 6D.
(0.15 MB TIF)

Figure S6 Proposed role for cohesin in restricting damage-induced recombination to sister chromatids in G2 cells, preventing homologous chromosome events and LOH. Presented are diagrams for damage-induced recombination in G_1_ and G_2_ cells. For clarity, events are shown in diploid cells; however, the concepts extend to tetraploid cells. While gene conversion between homologous chromosomes in G_1_ cells can lead to homozygosis over a short region, neither gene conversion nor crossing-over would lead to extended LOH. In G_2_ cells, gene conversion and/or crossing-over between sister chromatids does not change the genetic makeup of cells. However, recombination between homologous chromosomes can lead to localized changes as found for G_1_ cells, while crossing-over would lead to LOH, depending on segregation of the sister chromatids at mitosis. By holding sister chromatids together, cohesin could direct damage-induced recombination and repair towards sisters thereby preventing genetic instability.
(0.74 MB TIF)

Figure S7 Generation of *TYR1^+^* recombinants by reciprocal exchange between *TYR1* heteroalelles in tetraploid cells. Reciprocal exchange (RE) between homologous chromosomes can occur before (G_1_) or after (G_2_) replication of the *TYR1* locus. For the case of G_2_ cells, half the Tyr^+^ (*TYR1*) cells that underwent reciprocal exchange would have the “*y*” allele and half would not, assuming equal segregation of the sister chromatids. For recombinants induced in G_1_, all *TYR1^+^*recombinants due to RE would contain the “*y*” allele.
(0.28 MB TIF)
