# Supplementary figures and images for: Cohesin Is Limiting for the Suppression of DNA Damage–Induced Recombination between Homologous Chromosomes
Source: PLoS Genet. 2010 Jul 1;6(7):e1001006. doi: 10.1371/journal.pgen.1001006 (PMC2895640; doi:10.1371/journal.pgen.1001006)

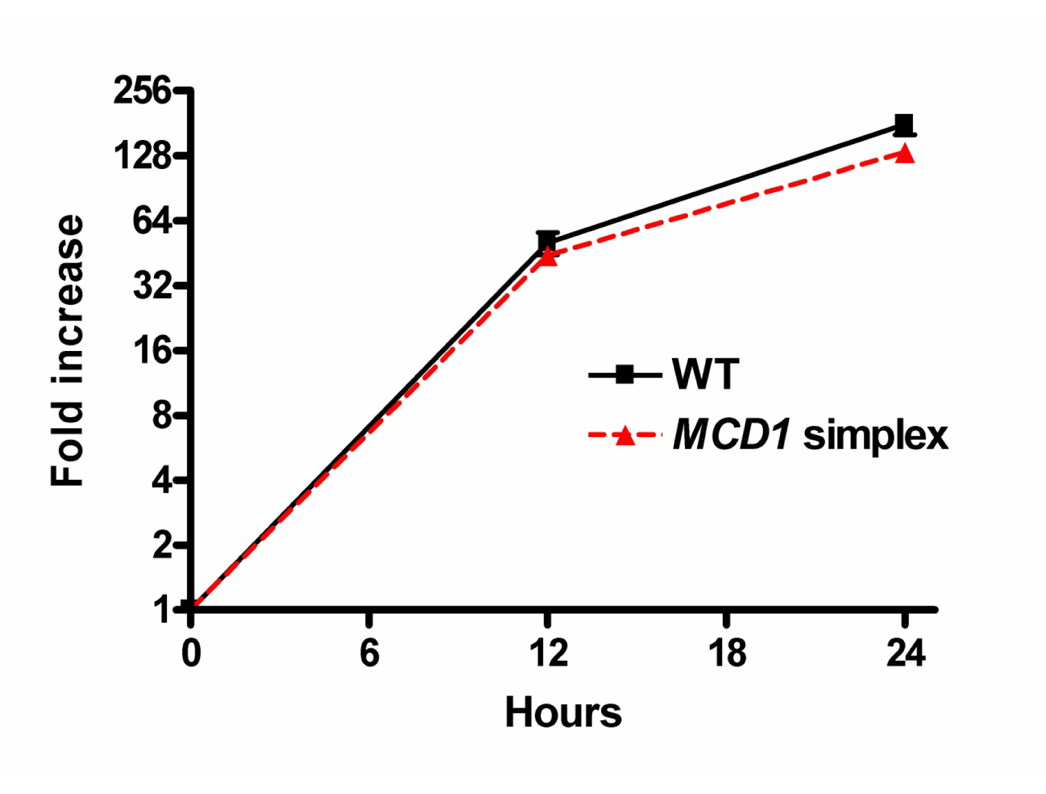

Supplement: Figure S1 — MCD1 simplex and WT (tetraploid) strains have comparable growth rates. Overnight cultures of WT and MCD1 simplex cells (2–4×10^7^ cells/ml) were diluted 100-fold into fresh YPDA medium Samples were collected, diluted and plated to YPDA after 12 and 24 hr. The culture density was calculated at each time point and the relative increase (compared to time “0”) was determined, results are combined from 4 different cultures of each genetic background. (0.20 MB TIF) [file pgen.1001006.s001.tif]

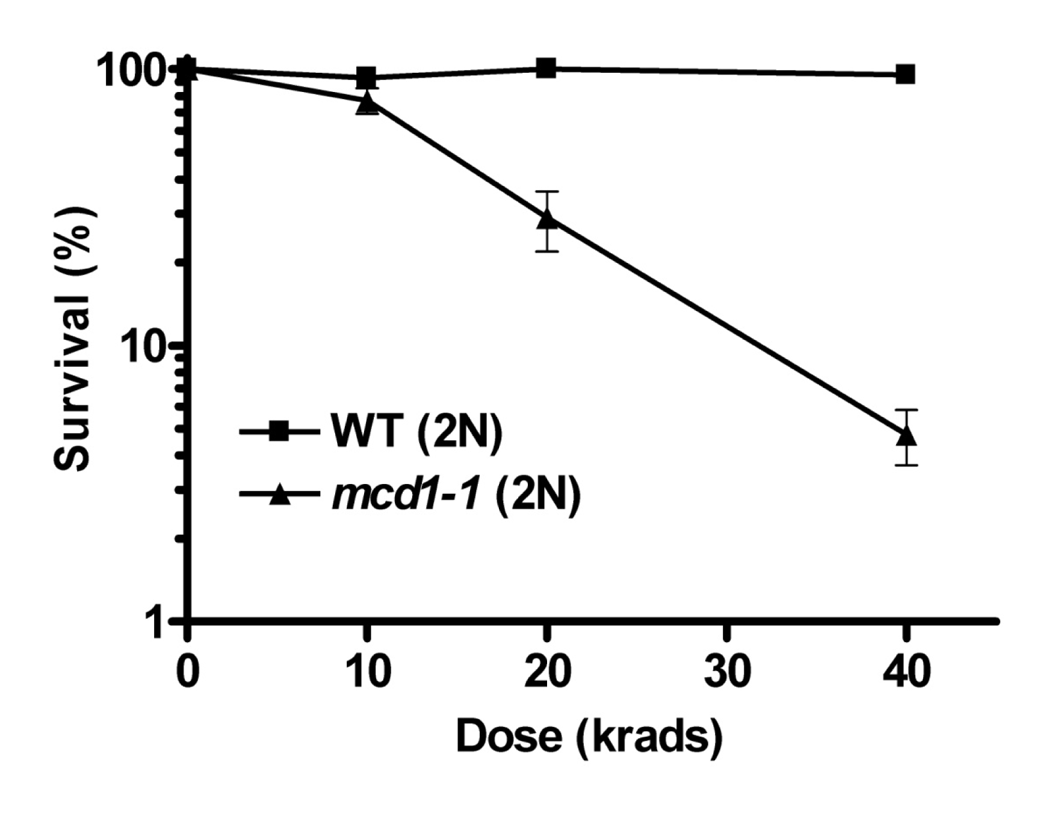

Supplement: Figure S2 — mcd1-1 diploids are sensitive to IR at semi-permissive temperature. The temperature sensitive mcd1-1 diploid cells were grown at permissive temperature (23°C) and arrested at G2/M with nocodazole for 3 hr. Survival was determined for cells that were irradiated, plated to YPDA plates and incubated at semi-permissive temp (32°C). The plating efficiency without irradiation of mcd1-1 at 32°C was 35% of that at 23°C. The same procedure was used with a WT diploid strain where no differences in plating efficiencies between 32° and 23°C were observed. Results are combined from 6 cultures of each genetic background. (0.17 MB TIF) [file pgen.1001006.s002.tif]

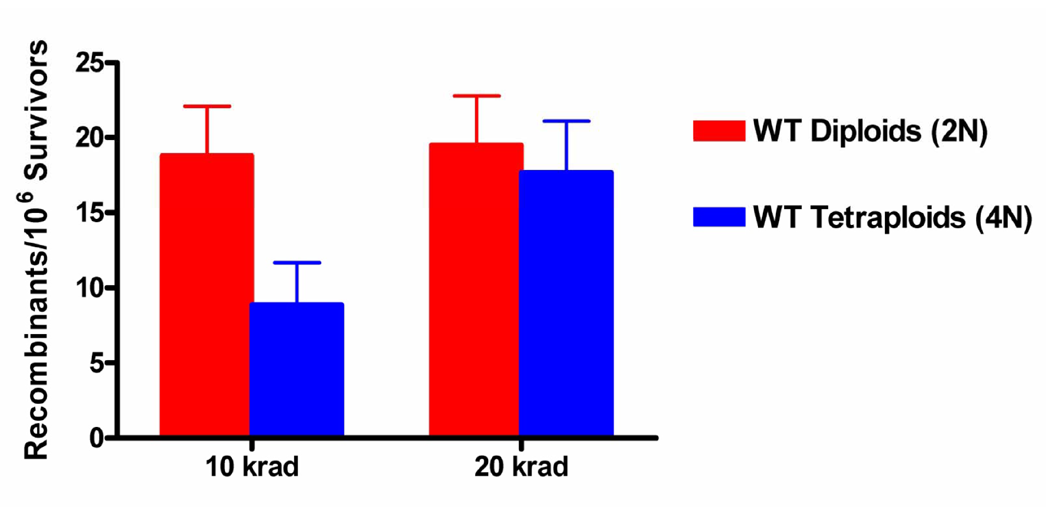

Supplement: Figure S3 — Recombination between homologous chromosome is similar for WT diploid and tetraploid cells arrested in G2/M. Cells were arrested with nocodazole as described in Figure 6 and Materials and Methods and irradiated with the indicated doses. The data for the tetraploid was taken from Figure 6B (at least 12 cultures were analyzed). Six diploid cultures were analyzed. (0.16 MB TIF) [file pgen.1001006.s003.tif]

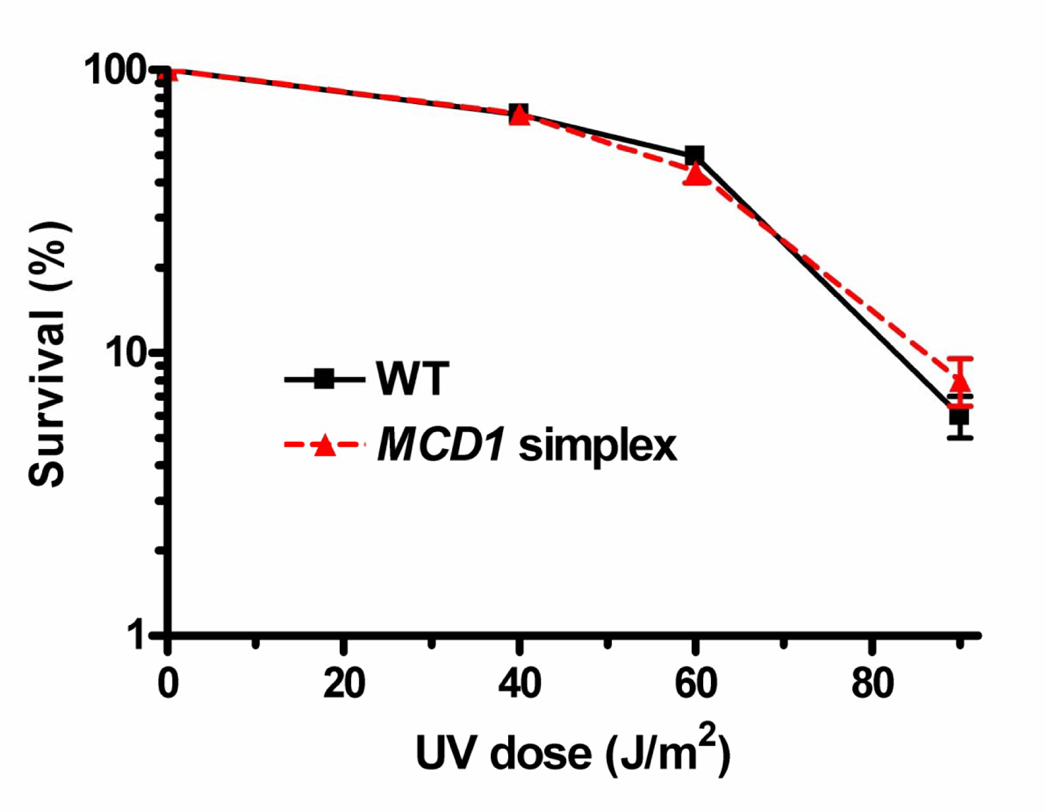

Supplement: Figure S4 — MCD1 simplex and WT strains exhibit similar UV sensitivity in asynchronous irradiated culture. Six late logarithmically growing cultures (2–4×10^7^ cells/ml) of WT and MCD1 simplex cells were diluted 1∶20,000 and pronged using a pronging that delivers1 µl per drop and 121 drops per plates on YPDA plates (described online at http://m.pu.ru/images/stories/Perfect%20order%20plating.html). Cells were irradiated at the indicated doses and colonies were counted after 3 days. (0.22 MB TIF) [file pgen.1001006.s004.tif]

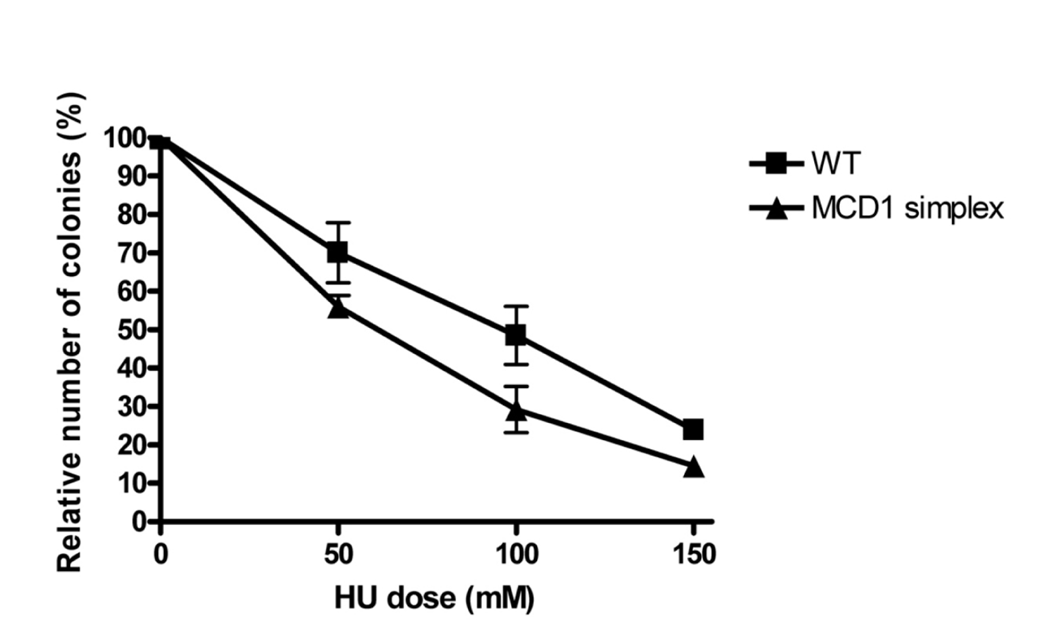

Supplement: Figure S5 — Hydroxurea induced growth inhibition of WT and MCD1 simplex strains. Stationary cultures were diluted to fresh YPDA medium and grown for 3 hr. The culture was divided into 4 equal parts and HU was added to a final concentration of 0, 50, 100 or 150 mM. Cells were grown overnight in the presence of HU then collected, diluted and spread on to synthetic complete media. Relative growth inhibition was determined from the number of colonies arising after the various treatments. This was the same procedure used to determine TYR1 recombination, described in Figure 6D. (0.15 MB TIF) [file pgen.1001006.s005.tif]

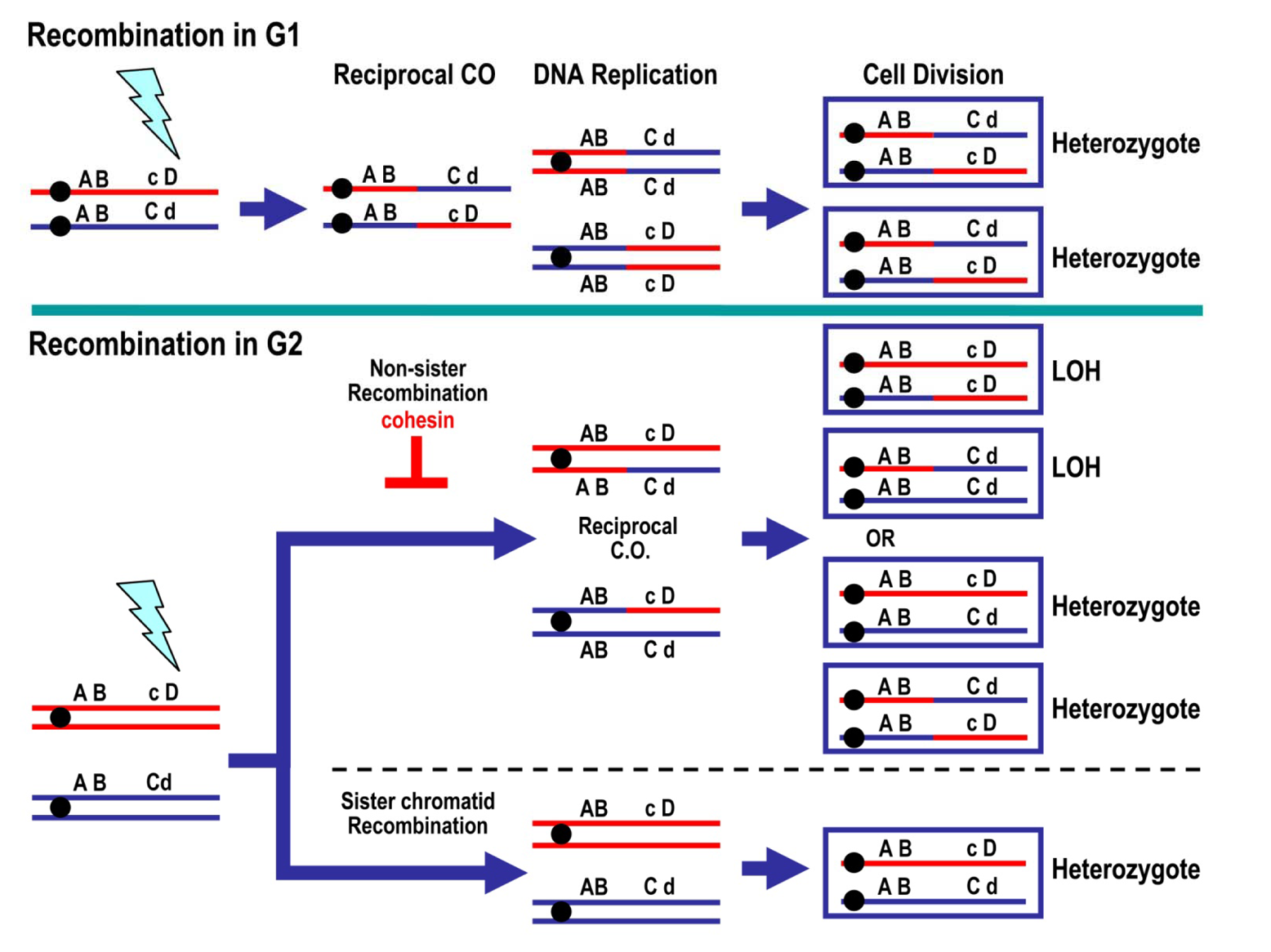

Supplement: Figure S6 — Proposed role for cohesin in restricting damage-induced recombination to sister chromatids in G2 cells, preventing homologous chromosome events and LOH. Presented are diagrams for damage-induced recombination in G1 and G2 cells. For clarity, events are shown in diploid cells; however, the concepts extend to tetraploid cells. While gene conversion between homologous chromosomes in G1 cells can lead to homozygosis over a short region, neither gene conversion nor crossing-over would lead to extended LOH. In G2 cells, gene conversion and/or crossing-over between sister chromatids does not change the genetic makeup of cells. However, recombination between homologous chromosomes can lead to localized changes as found for G1 cells, while crossing-over would lead to LOH, depending on segregation of the sister chromatids at mitosis. By holding sister chromatids together, cohesin could direct damage-induced recombination and repair towards sisters thereby preventing genetic instability. (0.74 MB TIF) [file pgen.1001006.s006.tif]

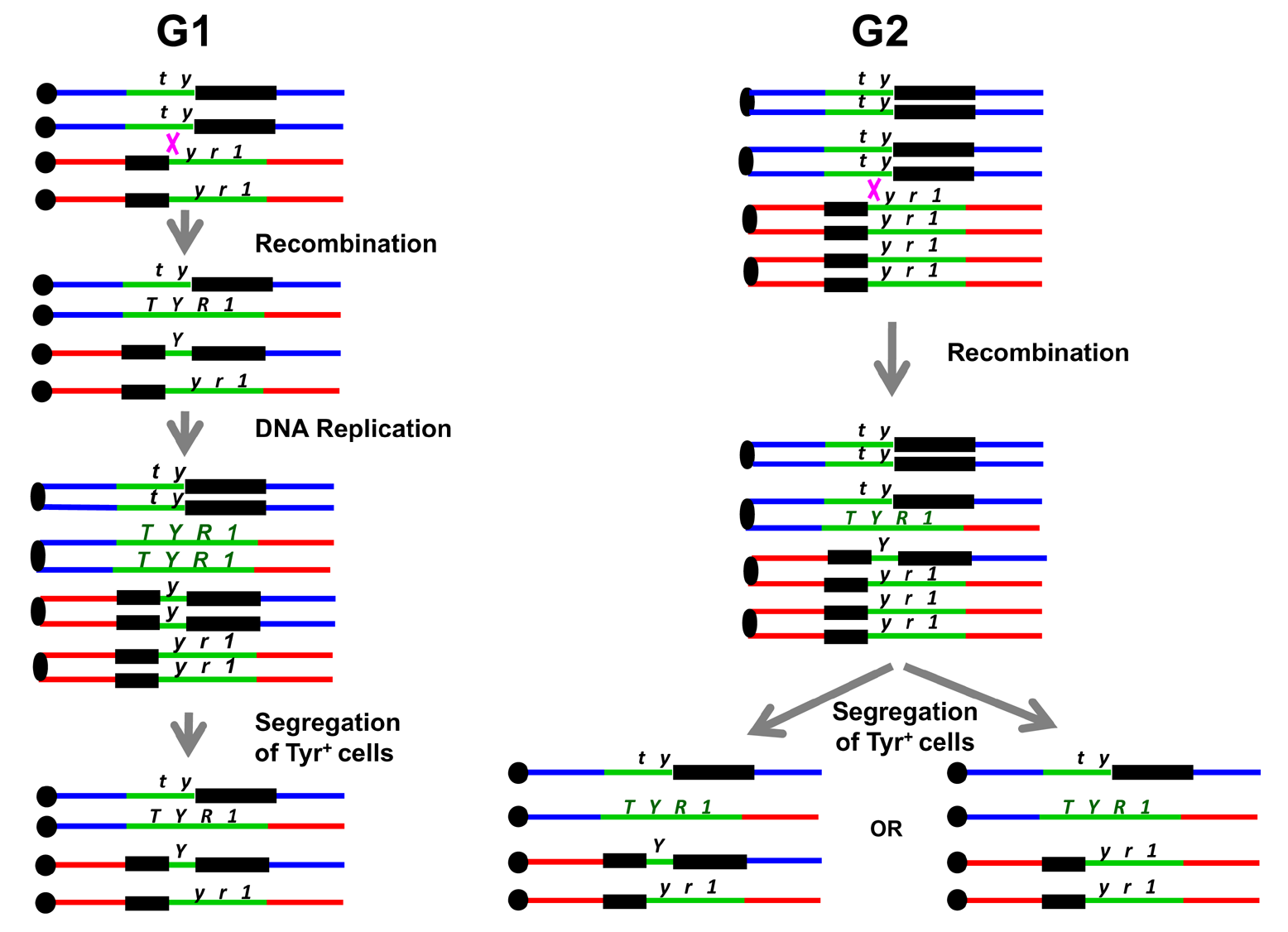

Supplement: Figure S7 — Generation of TYR1+ recombinants by reciprocal exchange between TYR1 heteroalelles in tetraploid cells. Reciprocal exchange (RE) between homologous chromosomes can occur before (G1) or after (G2) replication of the TYR1 locus. For the case of G2 cells, half the Tyr+ (TYR1) cells that underwent reciprocal exchange would have the “y” allele and half would not, assuming equal segregation of the sister chromatids. For recombinants induced in G1, all TYR1+recombinants due to RE would contain the “y” allele. (0.28 MB TIF) [file pgen.1001006.s007.tif]
